# Genomic Insights and Matrilineal Evolution Reveal Potential Dispersal Patterns of *Chitala* Species (Osteoglossomorpha: Notopteridae) in the Sundaland Biodiversity Hotspot

**DOI:** 10.1002/ece3.73257

**Published:** 2026-03-20

**Authors:** Flandrianto Sih Palimirmo, Angkasa Putra, Arif Wibowo, Sarifah Aini, Ah Ran Kim, Soo Rin Lee, Hye‐Eun Kang, Jung Hwa Choi, Kurniawan Kurniawan, Vitas Atmadi Prakoso, Indah Lestari Surbani, Hyun‐Woo Kim, Kyoungmi Kang, Shantanu Kundu

**Affiliations:** ^1^ Research Center for Biosystematics and Evolution, National Research and Innovation Agency, BRIN KST Ir. Soekarno West Java Indonesia; ^2^ Interdisciplinary Program of Marine and Fisheries Sciences and Convergent Technology Pukyong National University Busan Republic of Korea; ^3^ Research Center for Biota System, National Research and Innovation Agency, BRIN KST Ir. Soekarno West Java Indonesia; ^4^ Marine Integrated Biomedical Technology Center, National Key Research Institutes in Universities Pukyong National University Busan Republic of Korea; ^5^ Research Center for Marine Integrated Bionics Technology Pukyong National University Busan Republic of Korea; ^6^ Institute of Marine Life Science Pukyong National University Busan Republic of Korea; ^7^ Ocean and Fisheries Development International Cooperation Institute, College of Fisheries Science Pukyong National University Busan Republic of Korea; ^8^ National Research and Innovation Agency Jakarta Indonesia; ^9^ Diversitas Lestari Nusantara South Jakarta Indonesia; ^10^ Department of Marine Biology Pukyong National University Busan Republic of Korea; ^11^ Department of Biology, Faculty of Science and Technology Airlangga University Surabaya Indonesia; ^12^ International Graduate Program of Fisheries Science Pukyong National University Busan Republic of Korea

**Keywords:** ancient rivers, divergence time, mitochondrial DNA, old world fishes, phylogeny, Southeast Asia

## Abstract

The family Notopteridae is distributed across Africa and Asia and plays an important role in ecological functioning, local economies, and conservation initiatives. However, mitogenomic data for notopterids from the Sundaland hotspot remain scarce, limiting comprehensive understanding of their genetic architecture and matrilineal evolutionary history. To address this knowledge gap, the present study reports the first complete mitochondrial genome of *Chitala borneensis* and re‐sequences the mitogenome of *Chitala lopis* , two sympatric species endemic to Sundaland. Both *Chitala* mitogenomes exhibit the canonical teleost mitochondrial organization, comprising 37 genes and an AT‐rich control region, with *C. borneensis* (16,943 bp) representing the longest mitogenome reported within the genus to date. The codon usage analyses revealed a bias toward arginine, leucine, and serine, while nonsynonymous‐to‐synonymous substitution rate estimates indicated that most protein‐coding genes are evolving under purifying selection. The phylogenetic analyses recovered well‐supported African and Asian notopterid clades, with *C. borneensis* resolved as the sister lineage to *C. lopis* . Nevertheless, mitogenomic‐ and COI‐based phylogenetic frameworks inferred using Bayesian inference and maximum‐likelihood approaches supported multiple hypotheses regarding the systematic positions of mainland and island *Chitala* species in Southeast Asia. The divergence time estimates were congruent with historical biogeographic reconstructions, suggesting that Miocene tectonic events played a major role in the diversification of *Chitala* from other Asian notopterids. Furthermore, paleodrainage reorganization during the Last Glacial Maximum appears to have been critical in shaping the contemporary population structure of *Chitala* across the insular landscapes of this region. Collectively, this study provides new insights into the matrilineal evolutionary history of African and Asian notopterids, with particular emphasis on Sundaic *Chitala* species. The findings underscore the need for expanded taxon sampling and additional molecular data to improve population monitoring, inform conservation genetics, and ensure the long‐term persistence of these freshwater fishes within this biodiversity hotspot.

## Graphical abstract

This study reports the first complete mitochondrial genome of *Chitala borneensis* and the re‐sequencing of *Chitala lopis* from Sundaland. Analyses of genetic structure, matrilineal phylogenies, and TimeTree‐based divergence estimates elucidate the evolutionary history and dispersal dynamics of extant *Chitala* species. Our findings provide new insights into the roles of major geological events in shaping lineage diversification and highlight the influence of ancient paleodrainage systems on population structuring across both mainland and island ecosystems in Southeast Asia.

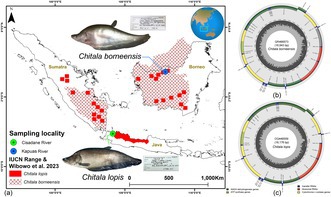

## 1 Introduction

The freshwater fishes represent a unique biotic component for biogeographical studies, as their distributions are constrained to discrete aquatic ecosystems and their evolutionary histories are often closely linked to continental drift and major geological events (Lundberg 1993; Chen et al. 2013; Matschiner et al. 2020). In this broader context, Southeast Asia is an under‐studied freshwater biodiversity hotspot with very high species diversity and many ancient lineages, making it an important region for studying historical biogeography and lineage diversification (Miller and Román‐Palacios 2021; Šlechtová et al. 2021). The featherbacks or knifefishes (family Notopteridae) demonstrate an Old‐World lineage of osteoglossomorph fishes that originated in the Early Cretaceous and subsequently diverged into two major lineages (Inoue et al. 2009). Currently, this family comprises 11 recognized species under four genera (*Papyrocranus*, *Xenomystus*, *Chitala*, and *Notopterus*), distributed across Africa, South Asia, and Southeast Asia (Fricke et al. 2026). They primarily inhabit tropical freshwater systems, where they serve key ecological functions and hold economic value as both food and ornamental fishes (Yanwirsal et al. 2017).

Specifically, the zoogeography of notopterids is striking, as the Asian representatives (two *Notopterus* species and six *Chitala* species) are distributed from Pakistan, India, Nepal, and Bangladesh, across Myanmar, Thailand, Laos, Cambodia, Vietnam, and Peninsular Malaysia, to the Indonesian islands (Sumatra, Java, and Borneo). However, the African taxa (two *Papyrocranus* species and a single *Xenomystus* species) are confined to West–Central Africa (Inoue et al. 2009; Lavoué et al. 2020; Ruzman et al. 2024). Within the genus *Chitala*, three species ( *C. blanci* , *C. chitala* , and *C. ornata* ) are distributed across South and mainland Southeast Asia, whereas three other congeners ( *C. borneensis* , *C. hypselonotus*, and *C. lopis* ) are restricted to Sundaland (Wibowo et al. 2024; Fricke et al. 2026). These distribution patterns offer a framework for investigating historical biotic connectivity between Africa and the Indian subcontinent, shaped by post‐breakup continental drift of the Madagascar–Seychelles–India landmass and subsequent Southeast Asian dispersal under the “Out‐of‐India” hypothesis (Ali and Aitchison 2008; Klaus et al. 2016; Yamahira et al. 2021). In addition, from a conservation standpoint, most notopterid populations are stable, yet several Southeast Asian taxa are experiencing rapid declines due to habitat loss, overfishing, and collection for the aquarium trade (IUCN 2026). Notably, *C. blanci* and *C. chitala* are currently listed as “Near Threatened,” while *C. lopis* is considered “Extinct,” with no confirmed records since the mid‐19th century (Chaudhry 2010; Vidthayanon 2011). The presumed extinction of *C. lopis* has been attributed to habitat degradation, overexploitation, and pollution in western and central Java (Ng 2020). However, the recent taxonomic reassessments of *C. lopis* in central Thailand not only refute its presumed “Extinct” status but also challenge its previously assumed endemicity to Sundaland (Musikasinthorn and Ngamtampong 2022).

Over the past 2 decades, molecular studies have increasingly contributed to clarifying notopterid systematics (Kumazawa and Nishida 2000; Lavoué and Sullivan 2004; Inoue et al. 2009). For instance, the occurrence of *C. lopis* in its type locality in West Java was recently confirmed using both morphological traits and partial mitochondrial COI sequences (Wibowo et al. 2023). Similarly, mitochondrial DNA markers (COI and Cytb) revealed the presence of three *Chitala* species ( *C. borneensis* , *C. lopis* , and *C. ornata* ) in Peninsular Malaysia (Ruzman et al. 2024). Despite these advances, substantial uncertainty persists regarding true species diversity due to inadequate molecular data, as most studies rely on partial loci and comprehensive genomic resources remain lacking. Although partial mitochondrial markers have proven useful, the complete mitochondrial genomes provide far greater resolution for studying phylogenetic relationships in freshwater fishes (Satoh et al. 2016; Izaki et al. 2025). To date, the complete mitogenomes have been reported for only four of the six recognized *Chitala* species ( *C. blanci* , *C. chitala* , *C. lopis* , and *C. ornata* ) (Inoue et al. 2009; Singh et al. 2019).

Although earlier mitogenomic studies have resolved the broader systematics of Notopteridae and clarified the divergence between African and Asian lineages (Inoue et al. 2009; Capobianco and Friedman 2024), substantial gaps remain in our understanding of evolutionary patterns within *Chitala* species. Specifically, ancient dispersal potentially facilitated by paleodrainage connectivity in shaping the evolutionary history and biogeographic patterns of the highly diverse *Chitala* lineages in Southeast Asia, particularly across the Sundaland biodiversity hotspot, remains poorly understood. Moreover, none of the previous studies have examined the mitogenomic characterization and structural variation of *Chitala* species, thereby limiting our understanding of their genetic diversity and matrilineal evolutionary dynamics. Beyond the reconstruction of phylogenetic relationships, divergence‐time estimation yields crucial inferences regarding the evolutionary history and spatiotemporal dispersal of taxa in the context of major geological and biogeographic events (Duchêne et al. 2014; Parham et al. 2012). Thus, estimating divergence times within the *Chitala* lineage is essential for elucidating their evolutionary trajectory and diversification patterns, particularly in the context of historical biogeographic processes of Southeast Asia.

In this context, the present study aims to: (i) generate the novel mitogenome of *C. borneensis* from the Kapuas River, Borneo; (ii) execute the mitogenomic characterization of *C. lopis* from the Cisadane River, Java; and (iii) evaluate mitogenome‐based phylogenetic relationships and divergence time estimation of Sundaic *Chitala* species within the broader Asian and African notopterid lineages. Collectively, these findings provide the first detailed mitogenomic characterization of two *Chitala* species, offering new insights into their maternal evolutionary history and dispersal pattern. Importantly, the mitogenomes generated in this study will enrich the genomic resources of this freshwater fish family and serve as valuable tools for future population and conservation genetic studies, thereby supporting more precise conservation strategies for these species across the Sundaland biodiversity hotspot.

## 2 Materials and Methods

### 2.1 Taxon Sampling and Morphological Investigation

**FIGURE 1:**
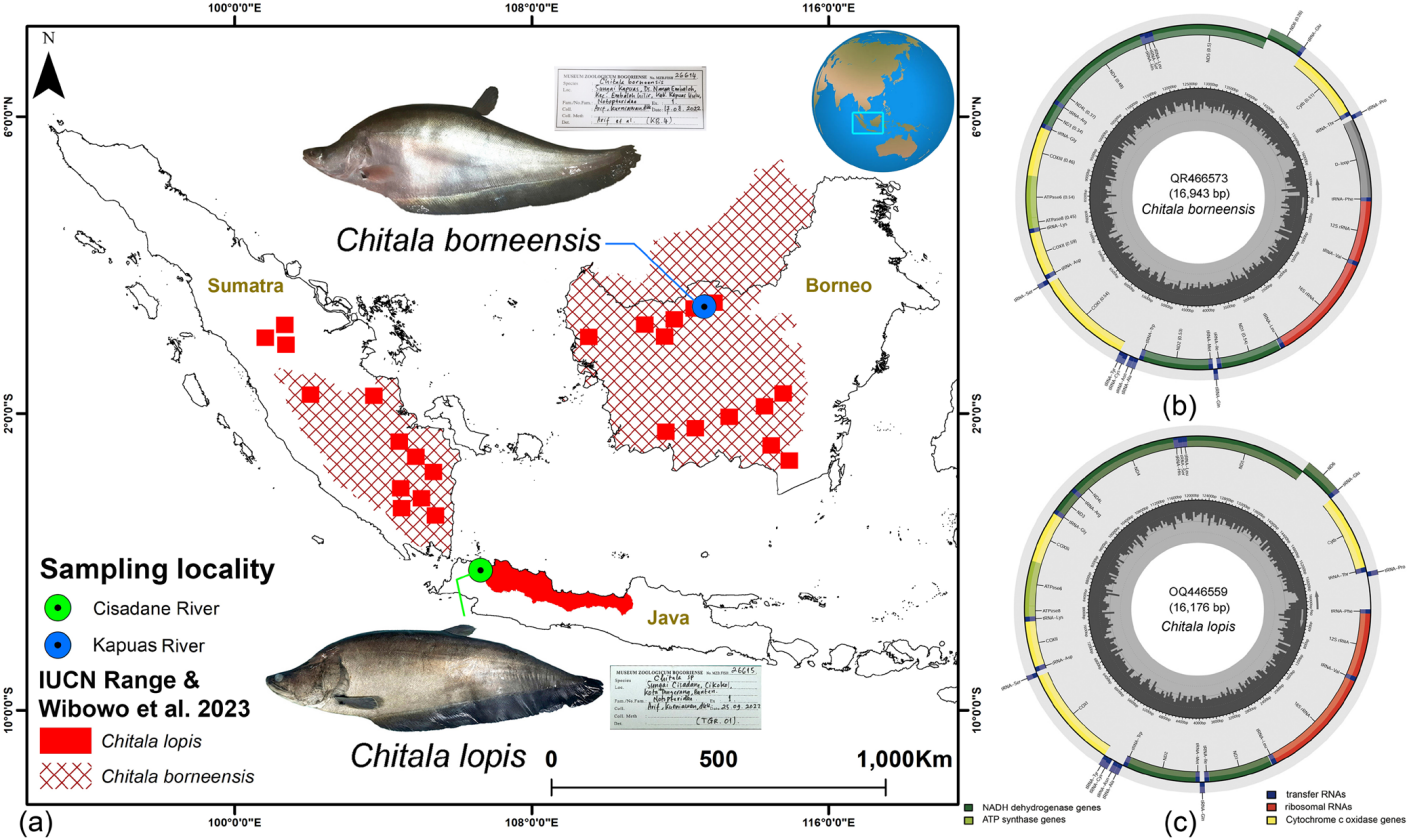
(a) Map showing the geographic distribution of *C. borneensis* and *C. lopis* across the three main islands of Indonesia, with blue and green markers indicating their respective collection localities. (b) The complete mitogenome of *C. borneensis* and (c) complete mitogenome of *C. lopis* were annotated and visualized using the MitoAnnotator online platform. The colored arcs represent PCGs, rRNAs, tRNAs, and the CR. The representative photographs of each species, along with their corresponding museum voucher information, were taken by the first author.

Two notopterid species were collected from two river systems within the Sundaland biodiversity hotspot, with *C. borneensis* from the Kapuas River in Borneo and *C. lopis* from the Cisadane River in Java (Figure 1a, Table S1). The sampling was conducted using cast nets, accompanied by the collection of collateral data such as geographic coordinates and field documentation. The species identification was validated as *C. borneensis* and *C. lopis* through combined morphological assessments and DNA barcoding, as reported previously (Wibowo et al. 2023). Representative vouchered specimens ( *C. borneensis* : MZB.FISH 26614; *C. lopis* : MZB.FISH 26615) from the Museum Zoologicum Bogoriense, Bogor, Indonesia (Wibowo et al. 2023), were selected for subsequent mitogenome sequencing (Figure 1a). The extracted tissue samples (~20 g) were deposited at the National Research and Innovation Agency (BRIN), Cibinong, Indonesia, as well as at the Molecular Physiology Laboratory, Department of Marine Biology, Pukyong National University, South Korea.

### 2.2 Ethics Statement

All specimen collection procedures followed the official guidelines of the Ministry of Marine Affairs and Fisheries, Republic of Indonesia (PER.10/MEN/2010 and Kepmen KP No. 1/2021), and the study was approved by the Agency for Marine and Fisheries Research and Human Resources, Ministry of Marine Affairs and Fisheries, Republic of Indonesia (2023/BRSDM/XII/2021). The molecular experiments were conducted in compliance with the relevant institutional guidelines and regulations approved by the host institution (Approval No. PKNUIACUC‐2025‐16). The collected specimen was euthanized by the direct addition of 2‐phenoxyethanol to the aquarium water at a concentration of 600 μL·L^−1^ (Nahon et al. 2017). After death was confirmed, the specimens were rinsed three times with Milli‐Q water (Merck‐Millipore, Molsheim, France) prior to molecular analysis. All experimental procedures were executed in accordance with the ARRIVE 2.0 guidelines (https://arriveguidelines.org) (Percie du Sert et al. 2020).

### 2.3 DNA Extraction and Next‐Generation Sequencing

Total genomic DNA was extracted using the Quick‐DNA MagBead Plus Kit (Zymo Research, Irvine, CA) according to the manufacturer's standard protocol. The quality and quantity of the extracted DNA were assessed using a NanoDrop spectrophotometer (Thermo Fisher Scientific D1000, WA, USA). To obtain complete mitogenomes of two *Chitala* species, the sequencing was performed using the NovaSeq platform (Illumina Inc., USA) at Macrogen (https://dna.macrogen.com/) in Daejeon, South Korea. The sequencing libraries were prepared following the manufacturer's instructions for the TruSeq Nano DNA High Throughput Library Prep Kit (Illumina Inc., USA). Briefly, 100 ng of genomic DNA was fragmented using adaptive focused acoustic technology (Covaris, USA), producing blunt‐ended double‐stranded DNA molecules with 5′ phosphorylation. Following end repair, the DNA fragments were size‐selected using a bead‐based method, an “A” base was added, and ligated with TruSeq DNA UD Indexing adapters. The resulting libraries were purified, PCR‐enriched, and quantified using qPCR (KAPA Library Quantification Kit), with quality assessment performed on an Agilent 4200 TapeStation D1000. The paired‐end sequencing (2 × 150 bp) produced over 20 million raw reads, which were subsequently processed with Cutadapt (http://code.google.com/p/cutadapt/) to remove adapter sequences and low‐quality bases (Phred *Q* score ≥ 20).

### 2.4 Mitogenome Assembly and Annotation

The mitogenomes of the two *Chitala* species were assembled from high‐quality paired‐end NGS reads using Geneious Prime v2023.0.1 (Kearse et al. 2012). The assembly was conducted through reference mapping against the mitogenome of a closely related species using the default mapping algorithms. The alignments of overlapping regions were manually inspected in MEGA v12 to ensure assembly accuracy (Kumar et al. 2024). The boundaries and orientations of the genes were validated using two independent annotation platforms, specifically Mitofish MitoAnnotator web server (https://mitofish.aori.u‐tokyo.ac.jp/annotation/input/) and the MITOS2 de novo annotation tool for metazoan mitochondrial genomes implemented in the Galaxy v1.1.6 web server (https://usegalaxy.eu) (Iwasaki et al. 2013; Al Arab et al. 2017; Donath et al. 2019). The protein‐coding genes (PCGs) were further confirmed by analyzing translated amino acid sequences with the Open Reading Frame Finder (https://www.ncbi.nlm.nih.gov/orffinder/) based on the vertebrate mitochondrial genetic code. The annotated mitogenomes of both *Chitala* species ( *C. borneensis* and *C. lopis* ) were deposited in GenBank, where they received unique accession numbers.

### 2.5 Mitogenomic Characterization and Comparative Analyses

The circular mitogenomic maps of the two newly assembled *Chitala* species were constructed using MitoAnnotator to facilitate genome visualization and annotation. A comparative analysis was subsequently performed to evaluate the genomic architecture and sequence variation of the two newly sequenced *Chitala* species in relation to seven previously reported representatives of six Asian notopterids (Table S1). The intergenic spacers separating adjacent genes and overlapping regions among *Chitala* species were manually calculated using Microsoft Excel v2016. The nucleotide compositions of PCGs, ribosomal RNA genes (rRNAs), and transfer RNA genes (tRNAs) were determined in MEGA v12. The base composition skews were calculated according to established formulas: AT‐skew = (A−T)/(A + T) and GC‐skew = (G−C)/(G + C) (Perna and Kocher 1995). The initiation and termination codons of each PCG were verified for conformity with the vertebrate mitochondrial genetic code in MEGA v12. The sliding window analysis of nucleotide diversity (*π*) among Asian notopterid species was conducted in DnaSP v6.0 using a window length of 200 bp and a step size of 25 bp (Rozas et al. 2017). The codon saturation in PCGs was assessed by visualizing patterns of transitions (s) and transversions (v) using DAMBE v6.0 (Xia 2017). The comparative analyses of nonsynonymous (Ka) and synonymous (Ks) substitution rates among Asian notopterid species were also performed in DnaSP v6.0. The codon usage metrics, including relative synonymous codon usage, amino acid composition, and codon distribution patterns within five *Chitala* species were further evaluated in DnaSP v6.0. In addition, the secondary structures of tRNAs in *C. borneensis* and *C. lopis* were predicted using tRNA and tmRNA detection via Aragorn on the Galaxy v1.1.6 web server (Laslett 2004).

### 2.6 Phylogenetic Inference

A total of 12 mitogenomes, including two newly sequenced and ten downloaded from public databases, representing nine notopterid species, were selected for phylogenetic dataset (Table S1). However, to evaluate notopterid lineages with all valid species, partial COI sequences of *Chitala hypselonotus* (Accession No. PP132012) and *Papyrocranus afer* (Accession No. HM882696) were also retrieved from GenBank and incorporated into an additional dataset to support the phylogenetic analyses. Both the mitogenome and partial COI of *Gnathonemus petersii* (AP008928), representing the family Mormyridae, were included as an outgroup. All 13 PCGs were concatenated using iTaxoTools v0.1 for mitogenome‐based phylogenetic analysis (Vences et al. 2021). The GTR + G + I model was selected as optimal based on the lowest Bayesian Information Criterion (BIC) calculated using PartitionFinder v2 (Lanfear et al. 2017). The Bayesian inference (BA) trees were reconstructed in MrBayes v3.1.2 using nst = 6, one cold and three hot MCMC chains, run for 10 million generations for the mitogenome dataset and 500,000 generations for the COI dataset, with the first 25% of samples discarded as burn‐in (Ronquist et al. 2012). The MCMC analysis was conducted until convergence was achieved, indicated by a standard deviation of split frequencies reaching 0.01 and the Potential Scale Reduction Factor (PSRF) for all parameters approaching 1.0. The maximum likelihood (ML) topologies were generated using PhyML v3.0, with branch support evaluated through 1000 bootstrap replicates (Guindon et al. 2010). Tree visualization for both BA and ML analyses was performed in iTOL v7 (https://itol.embl.de/login.cgi) (Letunic and Bork 2024).

### 2.7 Divergence Time Estimation

The divergence times in the present study were estimated using the RelTime method implemented in MEGA v12, which reduces the computational burden associated with BA analyses (Tamura et al. 2012; Mello et al. 2017; Mello 2018). The concatenated PCGs were analyzed using the previously inferred ML topology (.nwk file) as the reference tree, with appropriate outgroup taxa specified. Conceptually, divergence time estimation that uses the fossil record as primary calibration points is widely recommended in phylogenetic and paleontological studies, as it provides direct and independent temporal constraints (Duchêne et al. 2014; Parham et al. 2012). However, when relevant fossil records are sparse or unavailable, the use of secondary calibration points is often considered methodologically acceptable (Hipsley and Müller 2014; Schenk 2016). Accordingly, the divergence time analysis of notopterid evolutionary history in the current study employed two secondary calibration points derived from previous studies (Lavoué 2016; Capobianco and Friedman 2024). These calibration points correspond to: (i) the split between extant African and Asian knifefishes likely occurred during the Late Cretaceous (95% HPD = 104.3–60.6 Ma), well after the breakup of East and West Gondwana and (ii) the divergence between the Asian notopterid lineages (*Notopterus* vs. *Chitala*). However, no explicit minimum or maximum age constraints have been reported for the divergence between *Notopterus* and *Chitala* (Capobianco and Friedman 2024). Thus, to ensure consistency in divergence time analyses, a fixed maximum temporal bound was applied in both cases and implemented using the TimeTree framework in MEGA v12 (Ho et al. 2015; Mello 2018).

## 3 Results

### 3.1 Mitogenomic Structure and Organization

The circular mitogenomes of *C. borneensis* (Accession No. OR466573, 16,943 bp) and *C. lopis* (Accession No. OQ446559, 16,176 bp) comprise 13 PCGs, two rRNAs, 22 tRNAs, and a non‐coding AT‐rich control region (CR) (Figure 1b,c). Among them, 28 genes were located on the heavy strand, whereas one PCG (ND6) and eight tRNAs (trnQ, trnA, trnN, trnC, trnY, trnS2, trnE, and trnP) were located on the light strand (Table 1). The gene distribution on the heavy and light strands in *C. borneensis* and *C. lopis* matched that of other *Chitala* species, with mitogenome lengths ranging from 16,164 bp ( *C. ornata* ) to 16,943 bp ( *C. borneensis* ) (Table 2). The mitogenomes of *C. borneensis* and *C. lopis* exhibited seven and six gene‐overlapping regions totaling 34 bp and 25 bp, respectively, with the longest overlap (10 bp) in both species located between ATP8 and ATP6. Both *C. borneensis* and *C. lopis* mitogenomes also contained intergenic spacers totaling 66 bp, with 11 and 12 spacers respectively, and the longest spacer (34 bp) located between trnN and trnC in each species (Table S2). Furthermore, the nucleotide composition analysis revealed that the mitogenomes of *C. borneensis* and *C. lopis* had AT contents of 57.10% and 56.44%, respectively. Similar AT bias was observed in other *Chitala* mitogenomes, ranging from 56.48% in *C. lopis* (AP008922) to 57.37% in *C. ornata* (AP008923). The AT skew values for *C. borneensis* and *C. lopis* were 0.125 and 0.145, respectively, whereas the GC skew values were −0.296 and −0.314. The comparative mitogenome analysis across all *Chitala* species demonstrated the AT skew ranging from 0.126 in *C. ornata* to 0.146 in *C. lopis* , and the GC skew ranging from −0.315 in *C. lopis* to −0.289 in *C. blanci* (Table 2).

**TABLE 1:** Annotated genes with their boundaries, sizes, and intergenic nucleotide regions in the complete mitochondrial genomes of *Chitala borneensis* (OR466573)/ *Chitala lopis* (OQ446559).

Gene	Start	End	Strand	Size (bp)	Intergenic nucleotide	Start codon	Stop codon	Anti‐codon
*tRNA‐Phe (F)*	1	69	H	69	0	—	—	TTC
*12S rRNA*	70	1041/1042	H	972/973	0	—	—	—
*tRNA‐Val (V)*	1042/1043	1112/1113	H	71	0	—	—	GTA
*16S rRNA*	1113/1114	2813	H	1701/1700	0/1	—	—	—
*tRNA‐Leu (L2)*	2814/2815	2888/2889	H	75	9	—	—	TTA
*ND1*	2898/2899	3866/3867	H	969	2	ATG	TAA	—
*tRNA‐Ile (I)*	3869/3870	3939/3940	H	71	−1	—	—	ATC
*tRNA‐Gln (Q)*	3939/3940	4009/4010	L	71	−1	—	—	CAA
*tRNA‐Met (M)*	4009/4010	4077/4078	H	69	0	—	—	ATG
*ND2*	4078/4079	5122/5123	H	1045	0	ATG	T‐—	—
*tRNA‐Trp (W)*	5123/5124	5191/5192	H	69	1	—	—	TGA
*tRNA‐Ala (A)*	5193/5194	5261/5262	L	69	1	—	—	GCA
*tRNA‐Asn (N)*	5263/5264	5335/5336	L	73	34	—	—	AAC
*tRNA‐Cys (C)*	5370/5371	5435/5436	L	66	−1/0	—	—	TGC
*tRNA‐Tyr (Y)*	5435/5437	5505/5506	L	71/70	1	—	—	TAC
*COI*	5507/5508	7063/7056	H	1557/1549	−9/0	GTG	AGG/T‐—	—
*tRNA‐Ser (S2)*	7055/7057	7126/7128	L	72	4	—	—	TCA
*tRNA‐Asp (D)*	7131/7133	7202/7204	H	72	5	—	—	GAC
*COII*	7208/7210	7898/7900	H	691	0	ATG	T‐—	—
*tRNA‐Lys (K)*	7899/7901	7972/7974	H	74	1	—	—	AAA
*ATP8*	7974/7976	8141/8143	H	168	−10	ATG	TAA	—
*ATP6*	8132/8134	8814/8817	H	683/684	0/−1	ATG	TA−/TAA	—
*COIII*	8815/8817	9599/9601	H	785	0	ATG	TA—	—
*tRNA‐Gly (G)*	9600/9602	9670/9672	H	71	0	—	—	GGA
*ND3*	9671/9673	10,019/10021	H	349	0	ATG	T‐—	—
*tRNA‐Arg (R)*	10,020/10022	10,089/10091	H	70	0	—	—	CGA
*ND4L*	10,090/10092	10,386/10388	H	297	−7	ATG	TAA	—
*ND4*	10,380/10382	11,760/11762	H	1381	0	ATG	T‐—	—
*tRNA‐His (H)*	11,761/11763	11,830/11832	H	70	0	—	—	CAC
*tRNA‐Ser (S1)*	11,831/11833	11,897/11899	H	67	0	—	—	AGC
*tRNA‐Leu (L1)*	11,898/11900	11,970/11972	H	73	0	—	—	CTA
*ND5*	11,971/11973	13,809/13811	H	1839	−5	ATG	TAA	—
*ND6*	13,805/13807	14,326/14328	L	522	0	ATG	AGA	—
*tRNA‐Glu (E)*	14,327/14329	14,394/14396	L	68	5	—	—	GAA
*Cytb*	14,400/14402	15,540/15442	H	1141	0	ATG	T‐—	—
*tRNA‐Thr (T)*	15,541/15543	15,613/15615	H	73	3/2	—	—	ACA
*tRNA‐Pro (P)*	15,617/15618	15,686/15687	L	70	0	—	—	CCA
Control region	15,687/15688	16,943/16176	H	1257/489	—	—	—	—

**TABLE 2:** Nucleotide composition of complete mitochondrial genomes and different genes across *Chitala* species.

Species name	GenBank accession number	Size (bp)	A%	C%	G%	T%	A + T%	AT‐skew	GC‐skew
**Complete mitogenome**									
*C. borneensis*	OR466573	16,943	32.13	27.79	15.11	24.97	57.10	0.125	−0.296
*C. lopis*	OQ446559	16,176	32.31	28.62	14.94	24.12	56.44	0.145	−0.314
*C. lopis*	AP008922	16,177	32.36	28.61	14.90	24.12	56.48	0.146	−0.315
*C. chitala*	ON764424	16,248	32.00	27.95	15.31	24.74	56.74	0.128	−0.292
*C. chitala*	KX894524	16,375	32.05	27.84	15.21	24.84	56.89	0.127	−0.293
*C. ornata*	AP008923	16,164	32.30	27.77	14.87	25.07	57.37	0.126	−0.303
*C. blanci*	AP008921	16,272	32.15	27.73	15.30	24.80	56.96	0.129	−0.289
**PCGs**									
*C. borneensis*	OR466573	11,427	29.98	28.71	14.55	26.75	56.73	0.057	−0.327
*C. lopis*	OQ446559	11,420	30.11	29.16	14.30	26.44	56.54	0.065	−0.342
*C. lopis*	AP008922	11,419	30.14	29.14	14.27	26.45	56.59	0.065	−0.342
*C. chitala*	ON764424	11,422	29.96	28.77	14.49	26.78	56.74	0.056	−0.330
*C. chitala*	KX894524	11,407	30.04	28.79	14.43	26.74	56.78	0.058	−0.332
*C. ornata*	AP008923	11,419	30.10	28.43	14.18	27.30	57.40	0.049	−0.334
*C. blanci*	AP008921	11,419	29.93	28.33	14.76	26.98	56.91	0.052	−0.315
**rRNAs**									
*C. borneensis*	OR466573	2673	35.28	25.22	20.09	19.42	54.70	0.290	−0.113
*C. lopis*	OQ446559	2673	35.20	26.15	20.01	18.63	53.83	0.308	−0.133
*C. lopis*	AP008922	2674	35.27	26.14	19.97	18.62	53.89	0.309	−0.134
*C. chitala*	ON764424	2658	34.91	24.76	20.32	20.02	54.93	0.271	−0.098
*C. chitala*	KX894524	2660	34.85	24.77	20.34	20.04	54.89	0.270	−0.098
*C. ornata*	AP008923	2674	35.45	24.87	19.82	19.86	55.31	0.282	−0.113
*C. blanci*	AP008921	2673	35.28	25.03	20.13	19.57	54.84	0.286	−0.109
**tRNAs**									
*C. borneensis*	OR466573	1554	29.92	21.11	21.88	27.09	57.01	0.050	0.018
*C. lopis*	OQ446559	1553	29.68	21.06	22.15	27.11	56.79	0.045	0.025
*C. lopis*	AP008922	1555	29.84	20.96	22.06	27.14	56.98	0.047	0.025
*C. chitala*	ON764424	1570	29.62	21.46	22.10	26.82	56.43	0.050	0.015
*C. chitala*	KX894524	1553	29.68	21.31	22.28	26.72	56.41	0.053	0.022
*C. ornata*	AP008923	1554	29.28	21.04	22.46	27.22	56.50	0.036	0.033
*C. blanci*	AP008921	1555	29.52	21.41	22.19	26.88	56.40	0.047	0.018

### 3.2 Features of Protein‐Coding Genes

The mitogenomes of *C. borneensis* and *C. lopis* contained 13 PCGs, spanning 11,427 bp (67.44% of the total length) and 11,420 bp (70.59%), respectively. In both mitogenomes, the shortest PCG was ATP8, spanning 168 bp, while the longest PCG was NAD5, with a length of 1839 bp (Table 1). The length of PCGs in other *Chitala* species ranged from 11,407 bp ( *C. chitala* , KX894524) to 11,422 bp ( *C. chitala* , ON764424). The PCGs of *C. borneensis* exhibited an AT‐bias of 56.73%, with an AT skew of 0.057 and a GC skew of −0.327. Conversely, the PCGs of *C. lopis* showed a slightly lower AT‐bias of 56.54%, with an AT skew of 0.065 and a GC skew of −0.342. Similarly, the mitogenomes of other *Chitala* species displayed AT‐bias values ranging from 56.59% in *C. lopis* to 57.40% in *C. ornata* , with corresponding the AT skew values ranging from 0.049 in *C. ornata* to 0.065 in *C. lopis* and the GC skew values ranging from −0.342 in *C. lopis* to −0.315 in *C. blanci* (Table 2). In the *C. borneensis* , most of the PCGs began with the ATG start codon, with the exception of COI, which initiated with a GTG codon. A similar pattern of initiation codons was observed across all PCGs in the *C. lopis* . Regarding stop codons, the *C. borneensis* mitogenome, conventional TAA termination codon was observed in four PCGs (ND1, ATP8, NAD4L, and NAD5), while seven PCGs featured incomplete stop codons (T‐‐/TA‐). Additionally, the COI and NAD6 genes terminated with the nonstandard stop codons AGG and AGA, respectively. In the *C. lopis* , the conventional TAA termination codon was found in five PCGs (ND1, ATP8, ATP6, NAD4L, and NAD5), while seven PCGs had incomplete stop codons (T‐‐/TA‐), and NAD6 terminated with the AGA stop codon (Table S3).

### 3.3 Substitution Patterns and Codon Usage of Asian Notopterids

**FIGURE 2:**
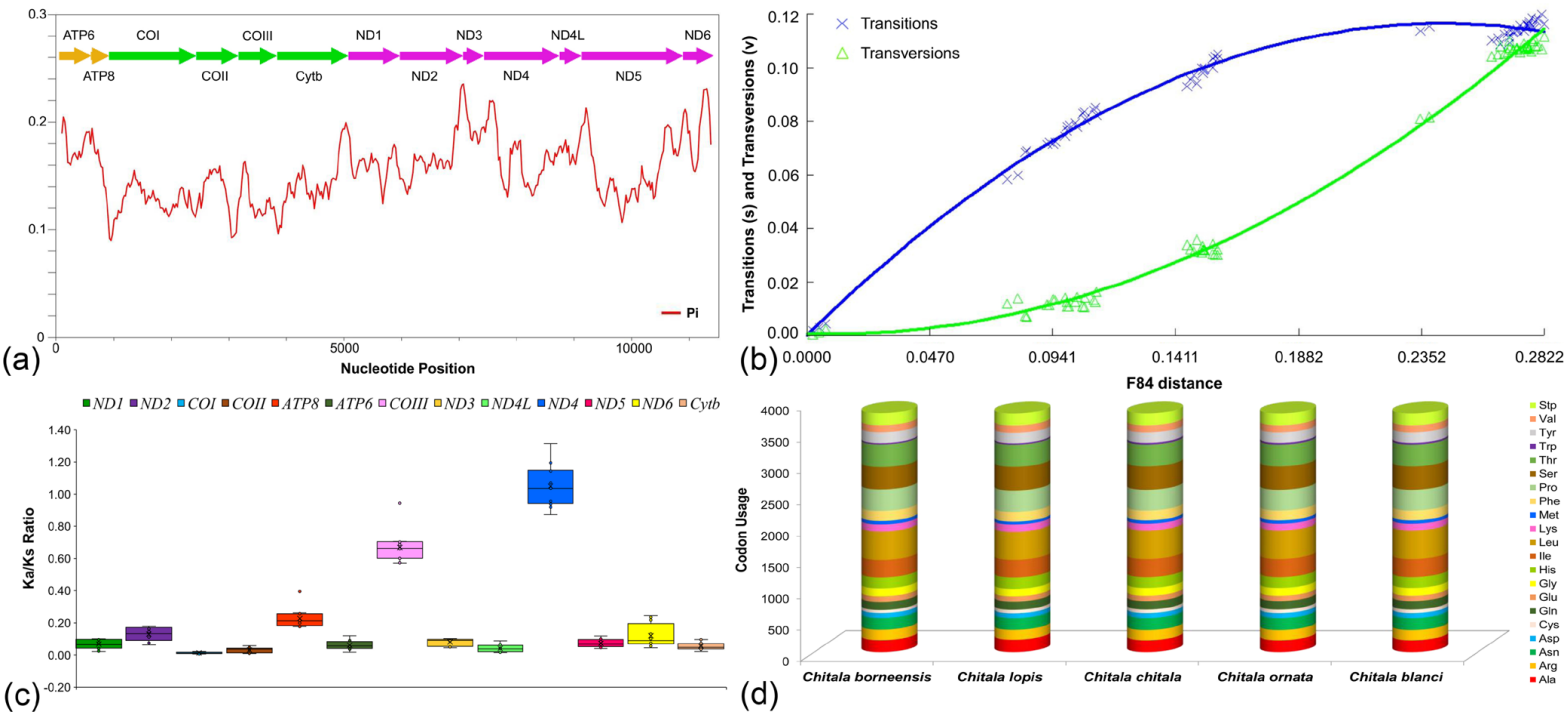
Genetic diversity and codon usage in Asian notopterid species. (a) The nucleotide diversity (*π*) across PCGs. (b) The scatter plots illustrating the relationship between transitions (s) and transversions (v) with genetic divergence in PCGs, based on FN84 distances. (c) The box plot of pairwise Ka/Ks ratios for each PCG. (d) Codon usage abundance in five *Chitala* species.

**FIGURE 3:**
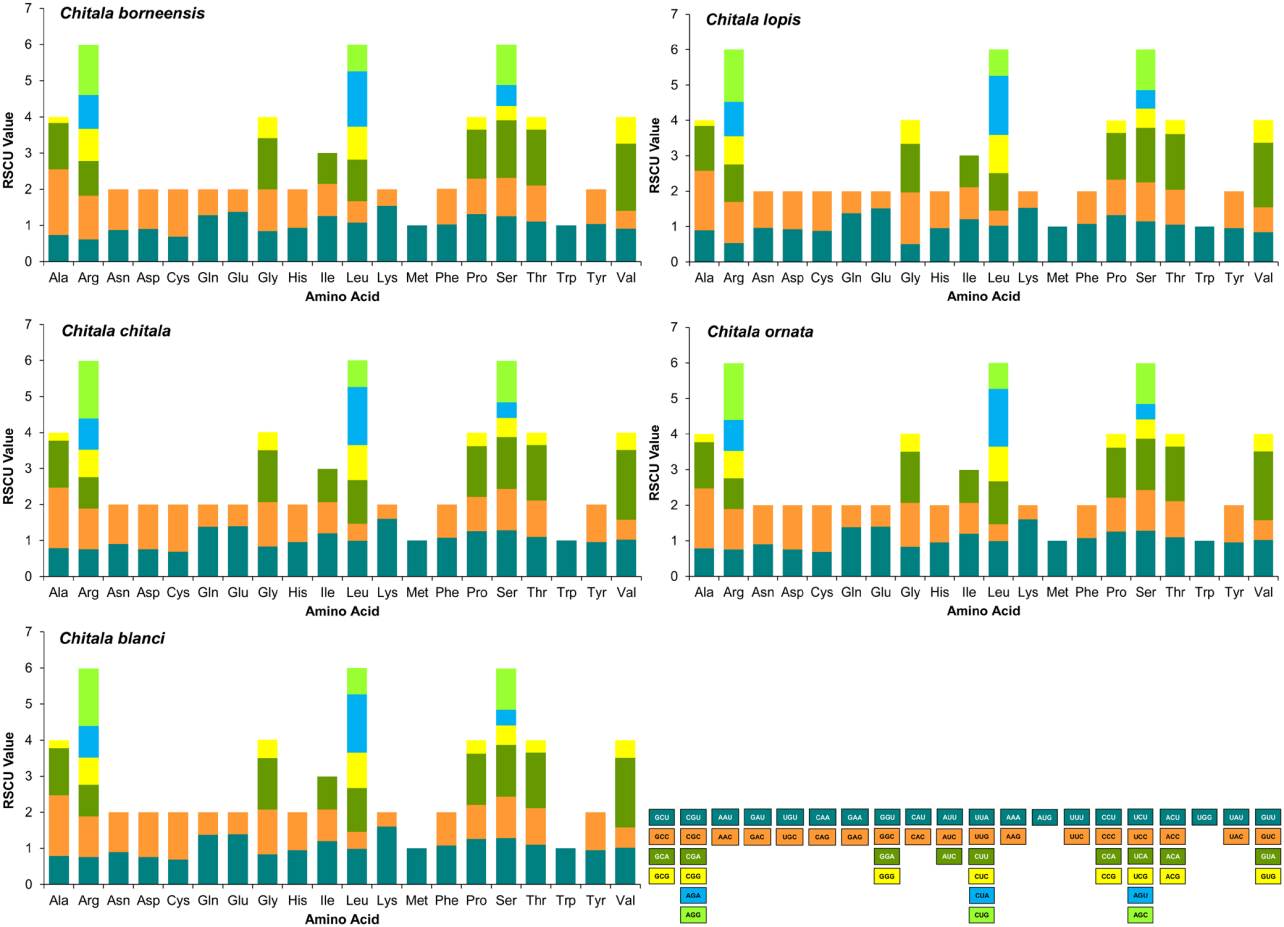
Comparative analysis of relative synonymous codon usage (RSCU) in five *Chitala* species, with codons grouped by amino acid on the *x*‐axis and RSCU values on the *y*‐axis.

The nucleotide diversity (*π*) across the assembled PCGs was assessed using a sliding window approach, revealing considerable variation among Asian notopterid species (*π* = 0.15421 across 4378 polymorphic sites) (Figure 2a). The sequence saturation analysis indicated that neither transitions (s) nor transversions (v) had reached saturation, as F84 divergence values continued to increase across the PCGs of Asian notopterid mitogenomes (Figure 2b). The Ka/Ks ratios ranged from 0.01293 ± 0.00491 for the COI gene to 1.04842 ± 0.07194 for the ND4 gene, following the order: COI < COII < ND4L < Cytb < ATP6 < ND1 < ND5 < ND3 < ND6 < ND2 < ATP8 < COIII < ND4 (Figure 2c; Table S4). The codon usage analysis further demonstrated that codons encoding arginine, leucine, and serine were most frequently utilized across the PCGs of five *Chitala* species, whereas the codons for methionine and tryptophan exhibited relatively lower usage frequencies (Figure 2d; Table S5). The relative synonymous codon usage analysis indicated that the codon GCG, which encodes alanine, was used significantly less frequently than its synonymous counterparts across the examined *Chitala* mitogenomes (Figure 3; Table S5).

### 3.4 Ribosomal RNA and Transfer RNA Genes

**FIGURE 4:**
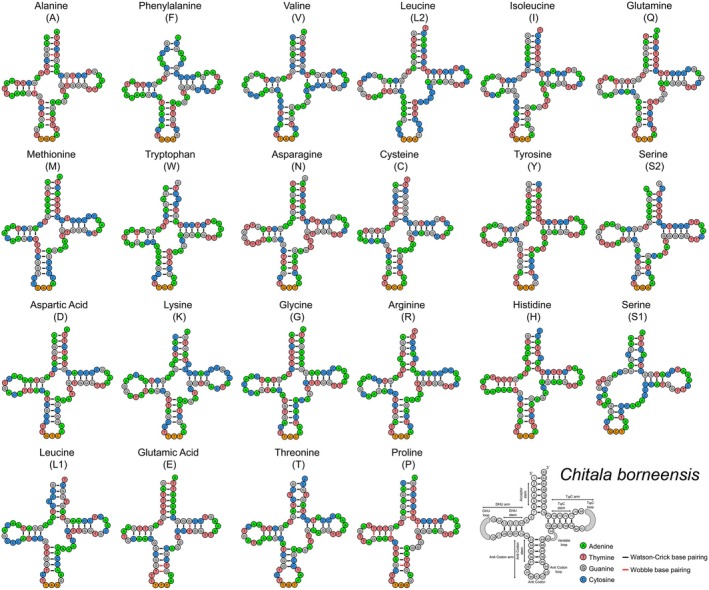
The secondary structures of 22 tRNAs in *C. borneensis* , labeled with full names and IUPAC‐IUB single‐letter amino acid codes, with the last structure showing nucleotide positions and stem‐loop details. The Watson‐Crick and wobble base pairings are indicated by black and red bars, respectively, highlighting structural variation.

**FIGURE 5:**
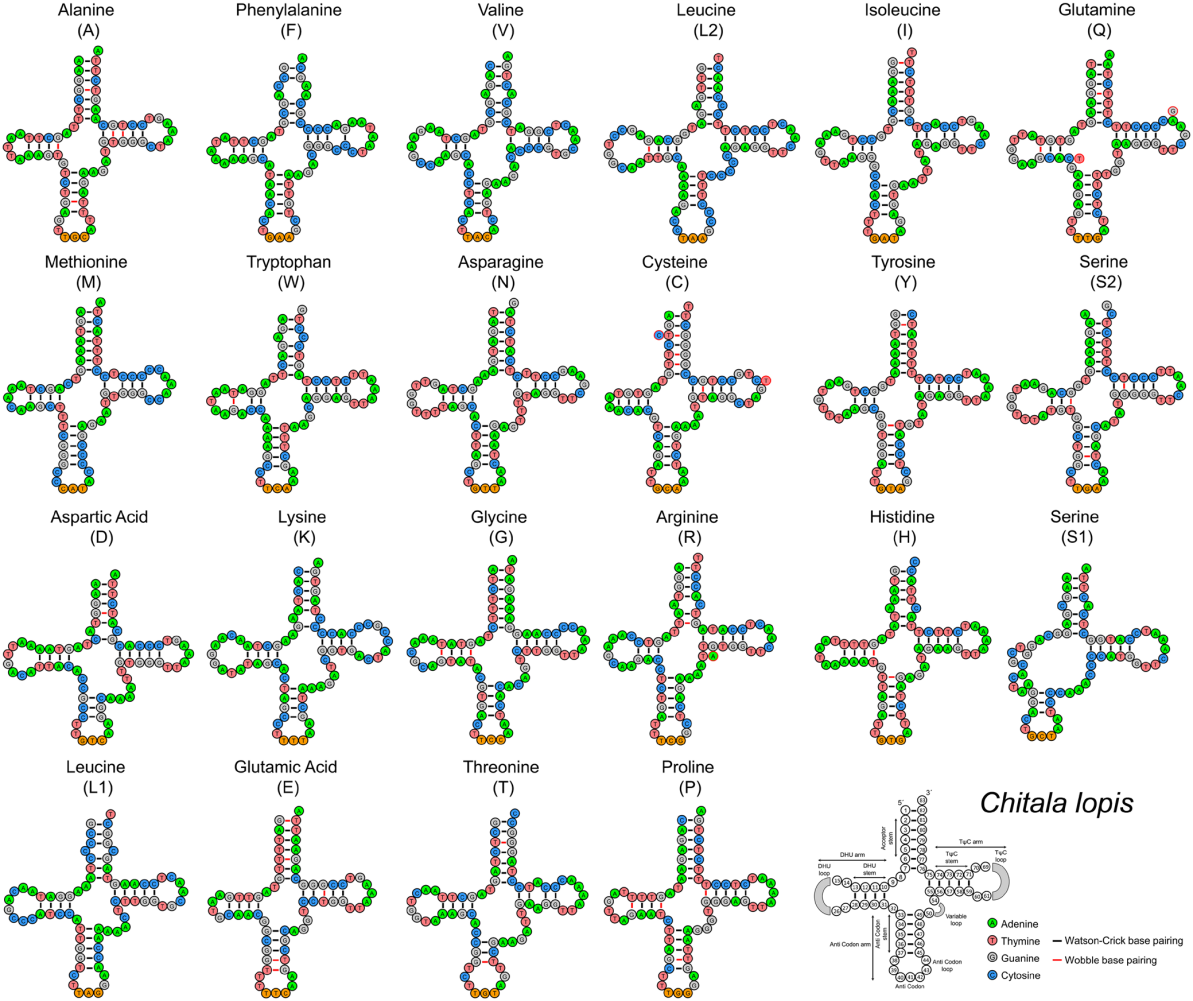
Secondary structures of 22 tRNAs in *C. lopis* , highlighting structural variation and alternative base pairing relative to the reference mitogenome (AP008922, marked by red circles). The Watson‐Crick and wobble base pairings are indicated by black and red bars, respectively.

In the mitogenomes of *C. borneensis* and *C. lopis* , the rRNA genes collectively spanned 2673 bp, representing 15.77% and 16.52% of the total mitogenome, respectively. These comprised a small ribosomal RNA (12S rRNA) of 972 bp and 973 bp, and a large ribosomal RNA (16S rRNA) of 1701 bp and 1700 bp in *C. borneensis* and *C. lopis* , respectively (Table 1). Among other *Chitala* species, the rRNA gene lengths ranged from 2658 bp in *C. chitala* to 2674 bp in *C. lopis* and *C. ornata* . The rRNA genes were AT‐biased, with AT contents ranging from 53.83% in *C. lopis* to 55.31% in *C. ornata* . The comparative analysis indicated the AT skew values between 0.270 in *C. chitala* and 0.309 in *C. lopis* , while the GC skew values ranged from −0.134 in *C. lopis* to −0.098 in *C. chitala* (Table 2). Additionally, both mitogenomes contained 22 tRNA genes, ranging from 66 bp (trnC) to 75 bp (trnL2) and comprising 9.17% ( *C. borneensis* ) and 9.60% ( *C. lopis* ) of the total mitogenome (Table 1). Across *Chitala* species, the tRNAs displayed AT bias ranging from 56.40% in *C. blanci* to 57.01% in *C. borneensis* , with the AT skew values ranging from 0.036 in *C. ornata* to 0.053 in *C. chitala* , and the GC skew values between 0.015 in *C. chitala* and 0.033 in *C. ornata* (Table 2). Most tRNAs of *C. borneensis* and *C. lopis* formed the canonical cloverleaf structure, except for trnS1, which lacked the dihydrouridine (DHU) stem. In the *C. borneensis* , 13 tRNAs (trnL2, trnQ, trnW, trnA, trnN, trnC, trnY, trnS2, trnD, trnG, trnH, trnE, and trnP) utilized both Watson–Crick and wobble base pairing, while the remaining nine relied exclusively on Watson–Crick pairing (Figure 4). In the *C. lopis* , 15 tRNAs (trnL2, trnI, trnQ, trnW, trnA, trnC, trnY, trnS2, trnD, trnG, trnR, trnH, trnE, trnT, and trnP) employed both types of base pairing, with the remaining seven using only Watson–Crick pairs. The comparative analysis with the GenBank *C. lopis* mitogenome (AP008922) further revealed alternative base pairing in trnQ, trnC, and trnR (Figure 5).

### 3.5 Matrilineal Phylogenetic Relationships

**FIGURE 6:**
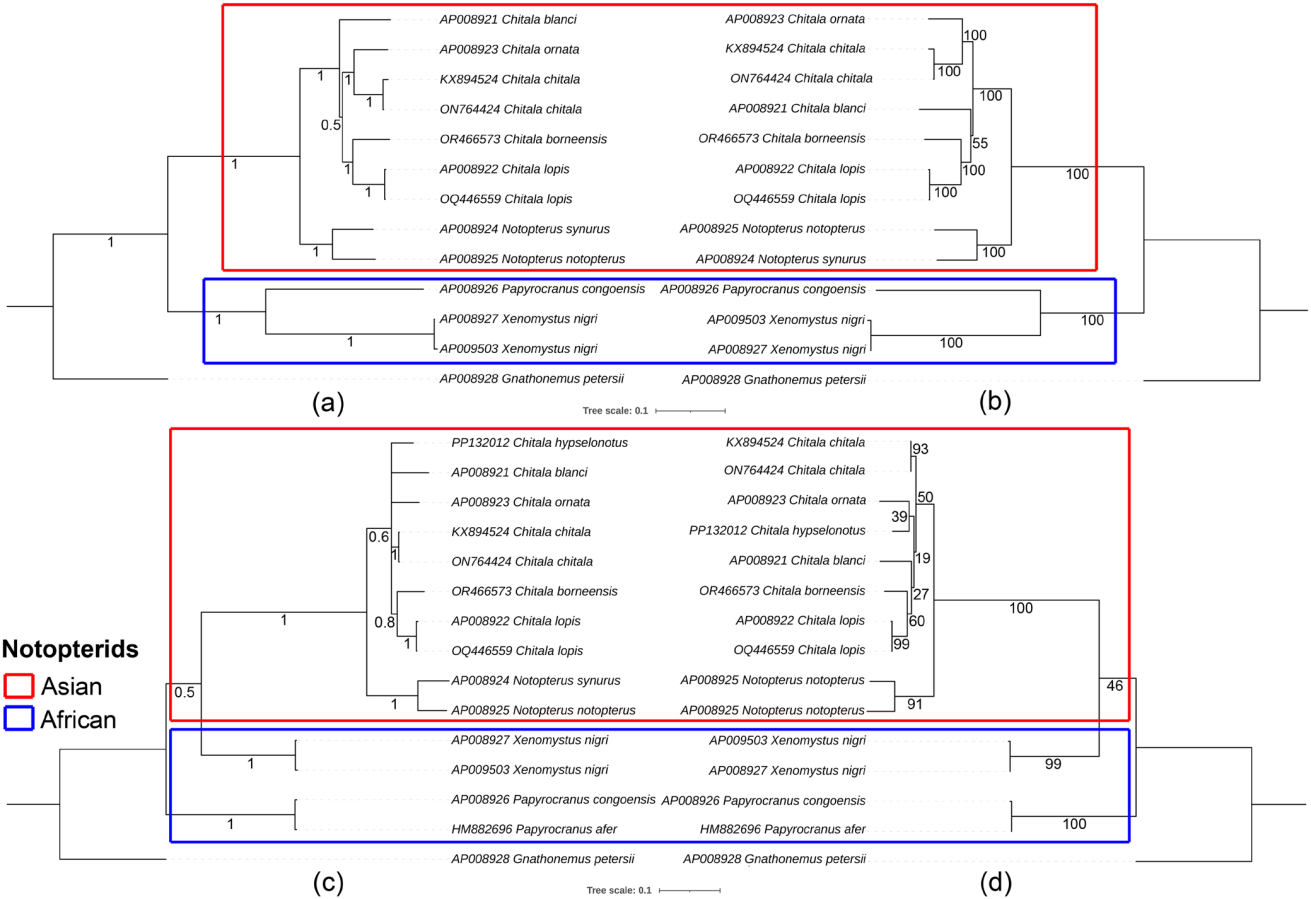
Matrilineal phylogenetic relationships among Asian and African notopterid species were inferred using complete mitochondrial genomes and partial COI sequences. The Bayesian inference (a, c) and maximum likelihood (b, d) trees were constructed using concatenated 13 PCGs (a, b) and partial COI sequences (c, d), with posterior probabilities (in black) providing statistical support at each node.

The matrilineal phylogenetic relationships among notopterid species were constructed using both complete mitogenomes and partial COI sequences, applying BA and ML methods (Figure 6a–d). Notably, two notopterid species, *C. hypselonotus* from Asia and *P. afer* from Africa, were included only in the COI phylogenetic analyses due to the unavailability of their mitogenomic data. Overall, all phylogenetic trees, whether based on mitogenomes or partial COI, consistently recovered two major geographic clades corresponding to African and Asian taxa. The African brown knifefish ( *Xenomystus nigri* ) formed a monophyletic group and clustered closely with other African notopterids of the genus *Papyrocranus*. Our analyses further supported the monophyly of two Asian subclades, comprising the genera *Chitala* and *Notopterus*, with high bootstrap and posterior probability values. The newly generated sequence of *C. borneensis* was recovered as sister lineage to *C. lopis* in both BA and ML phylogenies. Additionally, the newly sequenced specimen of *C. lopis* (OQ446559) clustered closely with a previously published Southeast Asian sequence (AP008922), confirming their intraspecific identity (Figure 6a–d). However, uniquely in the BA analysis of partial COI gene, a polytomy was observed among several *Chitala* species (*C. hypselonotus*, *C. blanci* , and *C. ornata* ) (Figure 6c).

### 3.6 Divergence Time of Notopterids

**FIGURE 7:**
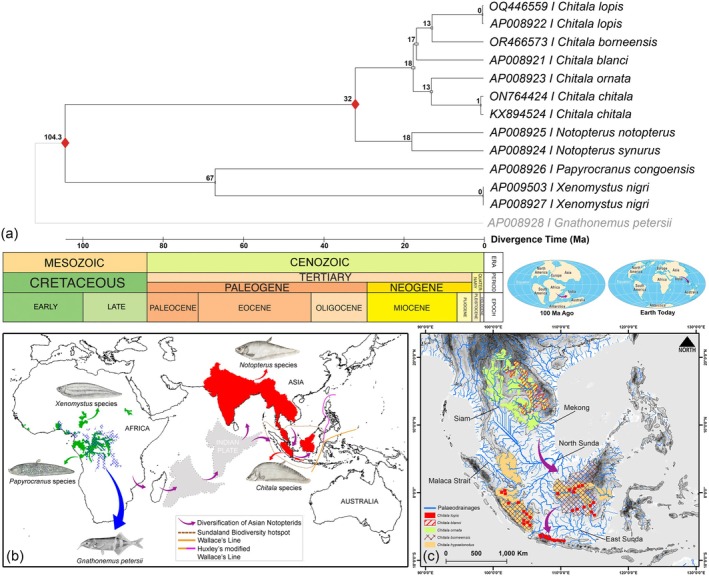
Evolutionary pattern and biogeographic interpretation of Asian notopterid species, with particular emphasis on *Chitala* within the Sundaland biodiversity hotspot. (a) TimeTree depicting estimated divergence times (Ma), with the red rhombus indicating the calibration node for both the split between African and Asian notopterids and the divergence between *Notopterus* and *Chitala* (Lavoué 2016; Capobianco and Friedman 2024). (b) Map illustrating the transcontinental diversification of notopterid species across Africa and Asia. Representative species photographs were obtained from the freely available Wikimedia Commons repository. (c) Map showing the hypothesized speciation routes of *Chitala* within Sundaland, likely driven by ancient river network dynamics during the Miocene. The distribution ranges of all *Chitala* species in Sundaland were obtained from the IUCN Red List of Threatened Species, with additional locality information for *C. lopis* incorporated from an earlier study (Wibowo et al. 2023). The paleodrainage networks of Sundaland were derived from the General Bathymetric Chart of the Oceans (GEBCO) 2025.

The RelTime method, implemented within a ML framework, provided estimates of divergence times for all internal nodes within Notopteridae species based on the current dataset. Hence, the TimeTree indicated that the split between African and Asian lineages occurred during the Cretaceous (104.3 Ma), concurrent with the tectonic separation of the Madagascar–Seychelles–India landmass from Africa (Figure 7a,b). Within the Asian clade, the divergence between the genera *Notopterus* and *Chitala* occurred during the Oligocene (32 Ma). The present divergence time estimation also suggested that the two African genera, *Papyrocranus* and *Xenomystus*, diverged during the Eocene (~67 Ma). Within the genus *Chitala*, divergence between mainland species ( *C. blanci* , *C. ornata* , and *C. chitala* ) and island species ( *C. borneensis* and *C. lopis* ) occurred between ~17 and ~18 Ma during the Miocene. Notably, the focal species of this study, *C. borneensis* and *C. lopis* , also diverged during the Miocene (~13 Ma). In the genus *Notopterus*, the split between South Asian *N. synurus* and Southeast Asian *N. notopterus* was estimated at ~18 Ma, closely aligned with the divergence between mainland and island *Chitala* species (Figure 7a).

## 4 Discussion

### 4.1 Mitogenomic Insights

This study reveals a comprehensive mitogenomic characterization of *Chitala* species, uncovering lineage‐specific structural, compositional features, and evolutionary dynamics within the family Notopteridae. The consistent nucleotide composition and pronounced AT‐bias, alongside observed genetic variation, likely reflect lineage‐specific mutational pressures influencing mitochondrial function, a pattern reported also in other teleosts (Kundu et al. 2024; Aini, Rina, et al. 2025). The strand‐specific skew patterns observed in *C. borneensis* and *C. lopis* suggest evolutionary constraints that may influence the efficiency and directionality of mitochondrial replication and transcription (Satoh et al. 2016). The frequent occurrence of incomplete stop codons in *Chitala* species is indicative of post‐transcriptional polyadenylation in PCGs (Izaki et al. 2025). The tRNA genes of *C. borneensis* and *C. lopis* display highly conserved structural features with minor interspecific variation, reflective of rigorous functional constraints and their critical roles in protein synthesis and genome integrity (Sebastian et al. 2022). Moreover, the absence of the DHU stem in trnS1 and the presence of wobble base pairing in multiple tRNAs in these two *Chitala* species may represent adaptive mechanisms that preserve mitochondrial translational efficiency (Yoon et al. 2024).

The nucleotide diversity analyses of Asian notopterid species illuminate the substantial intraspecific variation, suggesting complex evolutionary histories (Ord et al. 2023). The substitution saturation assessments further confirmed the retention of phylogenetic utility, validating these mitogenomes for evolutionary inference (Aini, Putra, et al. 2025). Across PCGs of Asian notopterid species, the Ka/Ks ratios were predominantly < 1, indicating purifying selection. In contrast, the ND4 gene showed the highest Ka/Ks ratio (1.048), which should be interpreted cautiously and may reflect relaxed purifying selection or stochastic effects rather than strong evidence of positive selection (Nei and Kumar 2000). The variation in Ka/Ks rankings among PCGs of Asian notopterids also underscores the functional constraints associated with mitochondrial energy metabolism (Ballantyne 2001). The codon usage patterns in five *Chitala* species may be influenced by nucleotide composition, translational efficiency requirements, and selective pressures (Sebastian et al. 2022). The predominant codons for arginine, leucine, and serine across five *Chitala* species presumably reflect their structural and energetic importance in mitochondrial proteins (Ballantyne 2001), whereas underrepresented codons, including methionine and tryptophan, may optimize translational accuracy and efficiency (Cope and Gilchrist 2022).

### 4.2 Matrilineal Evolutionary History of Notopterids

The evolutionary history of notopterids was initially inferred from morphological data (Nelson 1968; Hilton 2003). Subsequent molecular studies using partial mitochondrial markers, complete mitogenomes, and nuclear genes have refined phylogenetic understanding within these lineages (Inoue et al. 2009; Kumazawa and Nishida 2000; Lavoué and Sullivan 2004). Accordingly, the present mitogenome‐based phylogenetic analyses corroborate previous evolutionary investigations by revealing a clear biogeographic separation between African and Asian notopterid lineages (Inoue et al. 2009; Lavoué 2016; Capobianco and Friedman 2024). This dispersal history is also consistent with the geological framework of major tectonic events that enabled biotic exchange during the northward drift of the Indian Plate from Africa toward Eurasia (Ali and Aitchison 2008). The estimated divergence time indicates that African and Asian notopterids diverged during the Cretaceous period, subsequent to the separation of the Indian Plate from Africa (~130–160 Ma) (van Hinsbergen et al. 2011; Yoshida and Hamano 2015). These observations suggest that Asian notopterids likely originated from an ancestral lineage following the separation of the Indian Plate from Africa and subsequently experienced prolonged isolation. During this interval, the Indian landmass may have functioned as a “biotic ferry,” facilitating the evolution of Asian notopterids. Notably, the estimation of divergence time further indicates that the split between the Asian *Notopterus* and *Chitala* lineages occurred prior to the complete collision of the Indian Plate with Eurasia (~35–55 Ma). In addition, the subsequent diversification of several *Chitala* species during the Miocene likely reflects postcollision dispersal events across South and Southeast Asia. Collectively, these phylogenetic and temporal patterns support a post‐Gondwanan vicariance scenario driven by large‐scale tectonic processes, followed by episodes of secondary dispersal (Leroy et al. 2023). A similar biogeographic pattern has been well documented in other teleost lineages, which exhibit strong affinities between African and Asian fish assemblages (Capobianco and Friedman 2024; Yamahira et al. 2021).

### 4.3 Dispersal Patterns of *Chitala* Species in Sundaland

The present phylogenetic analyses of *Chitala* species largely corroborate previously proposed hypotheses and strongly support the close relationship between *C. lopis* and *C. borneensis* (Inoue et al. 2009; Wibowo et al. 2023). The present divergence‐time estimates indicate that these two species split during the Miocene and subsequently exhibited overlapping geographic distributions across the Indonesian islands of Sumatra and Borneo. Considering their divergence history and sympatric distribution, it is plausible that the overlapping ranges of both species were shaped by the paleodrainage systems of the North Sunda River network (de Bruyn et al. 2013; Sholihah et al. 2021). Furthermore, recent reports of *C. lopis* from the Pasak River in Central Thailand, together with its restricted distribution in Java (western and central river drainages), further support a historical biogeographic pattern influenced by the Siam–North Sunda and East Sunda river networks (Figure 7c). Nevertheless, the relationships among both mainland‐ and island‐distributed *Chitala* often appear intricate scenarios under the BA and ML frameworks, supporting alternative evolutionary hypotheses. In particular, the mainland species *C. blanci* is recovered either as the earliest‐diverging lineage among *Chitala* species in mitogenome‐based BA analyses, or as having diverged contemporaneously with *C. ornata* and *C. hypselonotus*, as depicted in COI‐based BA tree. In contrast, ML analyses based on both mitogenomic and COI data suggest that *C. blanci* diverged simultaneously with the island species *C. lopis* and *C. borneensis* from a shared common ancestor. Additionally, the phylogenetic placement of *C. hypselonotus*, inferred solely from partial COI sequences, varies among analytical approaches. The BA analyses yield a polytomy involving *C. hypselonotus*, *C. blanci* , and *C. ornata* , whereas ML analyses recover *C. hypselonotus* as the sister taxon to *C. ornata* . Accordingly, an alternative hypothesis is that *C. hypselonotus* represents a sister lineage to either *C. ornata* or *C. blanci* . Moreover, considering the fragmented distribution of *C. hypselonotus* across both the mainland and islands, it can also be inferred that the allopatric dispersal of its populations may have been facilitated by paleodrainage connections associated with the ancient Malacca Strait and the North Sunda river networks (Figure 7c).

Overall, both mitogenomic‐ and COI‐based phylogenetic topologies indicate that the evolutionary history of *Chitala* lineages in Southeast Asia, particularly within Sundaland, is more complex than a simple model of unidirectional ancestral dispersal. Furthermore, patterns of present‐day distribution and genetic connectivity in *Chitala* across the Indonesian archipelago suggest historical connectivity through extensive land bridges and paleoriver drainage networks (Voris 2000; Cheng and Faidi 2025). It is further evidence that, although pre–Last Glacial Maximum landscape connectivity likely promoted substantial gene flow, recent phylogeographic and population genetic analyses reveal distinct populations and cryptic diversity among *Chitala* and other freshwater fish species across Indonesian islands (Utami et al. 2022; Wibowo et al. 2023). This genetic diversity likely reflects prolonged geographic isolation and historical colonization driven by postglacial sea‐level rise and the fragmentation of paleodrainage systems, which helped maintain it at a regional scale.

### 4.4 Limitations and Recommendations

The present study reports the complete mitochondrial genomes of two *Chitala* species ( *C. borneensis* and *C. lopis* ) and examines their phylogenetic relationships and divergence times to infer historical biogeographic patterns, although several limitations should be acknowledged. To contextualize global notopterid lineages, partial mitochondrial COI sequences retrieved from GenBank, including those of Asian *C. hypselonotus* and African *P. afer* . Although these sequences provide preliminary support for phylogenetic inference, they are insufficient to fully resolve matrilineal evolutionary relationships. Consequently, future studies should prioritize generating complete mitogenomes for these two notopterid species to allow more robust reconstructions of their evolutionary history. Moreover, both the present analyses and previous studies on *C. lopis* and *C. borneensis* rely exclusively on mitochondrial markers, which reflect only maternal inheritance. Thus, to achieve a more comprehensive understanding of their evolutionary history, future research should integrate additional genetic markers, particularly nuclear genes and phylogenomic approaches. Therefore, exhaustive sampling of all *Chitala* species across South and Southeast Asia, coupled with multilocus phylogeographic analyses, would further clarify their true genetic diversity and population‐level dispersal patterns across mainland and insular ecosystems. Additionally, divergence time estimates in this study were inferred solely from notopterid lineages and are limited by reliance on only two secondary calibration points derived from previous studies and the exclusive use of mitochondrial data. Hence, future research should expand comprehensive molecular datasets across the Osteoglossomorpha lineage and incorporate additional calibration points, including primary fossil evidence, to obtain more precise and robust divergence time estimates. Finally, ongoing advances in the reconstruction of paleodrainage networks based on geological and hydrological data offer promising opportunities. Thus, integrating such refined paleodrainage models with molecular clock analyses will greatly enhance our ability to infer the evolutionary history and dispersal dynamics of this freshwater taxa within the insular ecosystems of the Sundaland biodiversity hotspot.

## 5 Conclusion

This study provides a comprehensive characterization of the mitochondrial genomes of *C. borneensis* and *C. lopis* , substantially expanding the available genetic resources for the family Notopteridae. Both mitogenomes exhibited conserved gene content and order, as well as an overall AT‐biased nucleotide composition. The phylogenetic analyses robustly supported the monophyly of Asian and African notopterids and resolved *C. borneensis* and *C. lopis* as sister taxa. The divergence time estimates were consistent with historical biogeographic reconstructions, suggesting that Miocene tectonic events drove the diversification of *Chitala* species from other Asian notopterids. Furthermore, the paleodrainage reorganization during the Last Glacial Maximum further played a critical role in shaping the present‐day population structure of *Chitala* within the insular habitats of Sundaland. Collectively, these findings offer valuable insights into the mitogenomic architecture and evolutionary dynamics of *Chitala*, establishing a robust molecular framework to support taxonomic resolution. Given the restricted distribution and heightened vulnerability of these species, expanded sampling and the generation of additional molecular data will be essential for effective population monitoring, informing conservation genetics, and ensuring the long‐term persistence of these freshwater fishes within the Sundaland biodiversity hotspot.

## Author Contributions

**Flandrianto Sih Palimirmo:** data curation (supporting), formal analysis (supporting), methodology (supporting), writing – original draft (supporting). **Angkasa Putra:** data curation (supporting), formal analysis (supporting), methodology (supporting), software (supporting), writing – original draft (supporting). **Arif Wibowo:** funding acquisition (equal), resources (supporting), supervision (supporting), validation (supporting). **Sarifah Aini:** methodology (supporting), software (supporting). **Ah Ran Kim:** software (supporting), visualization (supporting). **Soo Rin Lee:** investigation (supporting), visualization (supporting). **Hye‐Eun Kang:** formal analysis (supporting), investigation (supporting). **Jung Hwa Choi:** visualization (supporting), writing – review and editing (supporting). **Kurniawan Kurniawan:** data curation (supporting), validation (supporting). **Vitas Atmadi Prakoso:** methodology (supporting), validation (supporting). **Indah Lestari Surbani:** data curation (supporting), investigation (supporting). **Hyun‐Woo Kim:** conceptualization (equal), project administration (equal), resources (supporting), supervision (supporting), writing – review and editing (supporting). **Kyoungmi Kang:** funding acquisition (equal), validation (supporting), visualization (supporting), writing – review and editing (supporting). **Shantanu Kundu:** conceptualization (equal), project administration (equal), resources (supporting), supervision (supporting), writing – original draft (supporting), writing – review and editing (supporting).

## Funding

This research was supported by the “Global Bluefood Leadership Project (RS‐2025‐02373103),” funded by the Ministry of Oceans and Fisheries, Republic of Korea.

## Conflicts of Interest

The authors declare no conflicts of interest.

## Supporting information


**Data S1:** ece373257‐sup‐0001‐supinfo.docx.

## Data Availability

The data supporting the findings of this study are publicly available in the NCBI GenBank database (https://www.ncbi.nlm.nih.gov) under the accession numbers OR466573 and OQ446559.
