# Supplementary material for: Genomic Insights and Matrilineal Evolution Reveal Potential Dispersal Patterns of Chitala Species (Osteoglossomorpha: Notopteridae) in the Sundaland Biodiversity Hotspot
Source: Ecol Evol. 2026 Mar 20;16(3):e73257. doi: 10.1002/ece3.73257 (PMC13093427; doi:10.1002/ece3.73257)
Supplement: Supplementary file 1 — Data S1: ece373257‐sup‐0001‐supinfo.docx. [file ECE3-16-e73257-s001.docx]

**SUPPORTING INFORMATION**

**Table S1** The generated and GenBank mitogenomes of the Notopteridae species.

| **S1. No.** | **Species Name** | **GenBank**  **Accession**  **Number** | **Size**  **(bp)** | **Collection**  **Information** | **Reference** |
| --- | --- | --- | --- | --- | --- |
| 1. | *Chitala borneensis* | OR466573 | 16943 | Kapuas River, Borneo | This study |
| 2. | *Chitala lopis* | OQ446559 | 16176 | Cisadane River, Java | This study |
| 3. | *Chitala lopis* | AP008922 | 16177 | Southeast Asia | Inoue et al. 2009 |
| 4. | *Chitala chitala* | ON764424 | 16248 | Bangladesh | GenBank |
| 5. | *Chitala chitala* | KX894524 | 16375 | Teesta river, India | Singh et al. 2019 |
| 6. | *Chitala ornata* | AP008923 | 16164 | Southeast Asia | Inoue et al. 2009 |
| 7. | *Chitala blanci* | AP008921 | 16272 | Southeast Asia | Inoue et al. 2009 |
| 8. | *Notopterus notopterus* | AP008925 | 15854 | Southeast Asia (Thailand) | Inoue et al. 2009 |
| 9. | *Notopterus synurus* | AP008924 | 16030 | South Asia (India) | Inoue et al. 2009 |
| 10. | *Xenomystus nigri* | AP009503 | 16635 | Ouémé river, Bénin | Lavoue et al. 2011 |
| 11. | *Xenomystus nigri* | AP008927 | 16635 | Tropical Africa | Inoue et al. 2009 |
| 12. | *Papyrocranus congoensis* | AP008926 | 16823 | Congo basin, Central Africa | Inoue et al. 2009 |
| 13. | *Gnathonemus petersii* | AP008928 | 16687 | Nigeria (outgroup) | Inoue et al. 2009 |

**Table S2** The intergenic nucleotide regions in various *Chitala* species.

| **Genes** | ***C. borneensis* (OR466573)** | ***C. lopis* (OQ446559)** | ***C. lopis* (AP008922)** | ***C. chitala* (ON764424)** | ***C. chitala* (KX894524)** | ***C. ornata* (AP008923)** | ***C. blanci* (AP008921)** |
| --- | --- | --- | --- | --- | --- | --- | --- |
| *tRNA-Phe (F)* | 0 | 0 | 0 | 0 | 0 | 0 | 0 |
| *12S rRNA* | 0 | 0 | 0 | 0 | 0 | 0 | 0 |
| *tRNA-Val (V)* | 0 | 0 | 0 | 0 | 0 | 0 | 0 |
| *16S rRNA* | 0 | 1 | 0 | 0 | 0 | 0 | 0 |
| *tRNA-Leu (L2)* | 9 | 9 | 9 | 8 | 0 | 9 | 9 |
| *ND1* | 2 | 2 | 2 | 3 | 3 | 2 | 2 |
| *tRNA-Ile (I)* | -1 | -1 | -1 | -1 | -1 | -1 | -1 |
| *tRNA-Gln (Q)* | -1 | -1 | -1 | -2 | -1 | -1 | -1 |
| *tRNA-Met (M)* | 0 | 0 | 0 | -1 | 0 | 0 | 0 |
| *ND2* | 0 | 0 | 0 | -2 | 0 | 0 | 0 |
| *tRNA-Trp (W)* | 1 | 1 | 1 | -1 | 1 | 1 | 1 |
| *tRNA-Ala (A)* | 1 | 1 | 1 | 0 | 1 | 1 | 1 |
| *tRNA-Asn (N)* | 34 | 34 | 34 | 32 | 34 | 34 | 34 |
| *tRNA-Cys (C)* | -1 | 0 | -1 | -1 | -1 | -1 | -1 |
| *tRNA-Tyr (Y)* | 1 | 1 | 1 | 1 | 1 | 1 | 1 |
| *COI* | -9 | 0 | 0 | -2 | 0 | 0 | 0 |
| *tRNA-Ser (S2)* | 4 | 4 | 4 | 4 | 5 | 4 | 4 |
| *tRNA-Asp (D)* | 5 | 5 | 5 | 0 | 0 | 5 | 5 |
| *COII* | 0 | 0 | 0 | 0 | 0 | 0 | 0 |
| *tRNA-Lys (K)* | 1 | 1 | 1 | 0 | 1 | 1 | 1 |
| *ATP8* | -10 | -10 | -10 | -10 | -10 | -10 | -10 |
| *ATP6* | 0 | -1 | 0 | -1 | -1 | 0 | 0 |
| *COIII* | 0 | 0 | 0 | 0 | 0 | 0 | 0 |
| *tRNA-Gly (G)* | 0 | 0 | 0 | 0 | 0 | 0 | 0 |
| *ND3* | 0 | 0 | 0 | 0 | 0 | 0 | 0 |
| *tRNA-Arg (R)* | 0 | 0 | 0 | 0 | 0 | 0 | 0 |
| *ND4L* | -7 | -7 | -7 | -7 | -7 | -7 | -7 |
| *ND4* | 0 | 0 | 0 | 0 | 0 | 0 | 0 |
| *tRNA-His (H)* | 0 | 0 | 0 | 0 | 0 | 0 | 0 |
| *tRNA-Ser (S1)* | 0 | 0 | 0 | -1 | 0 | 0 | 0 |
| *tRNA-Leu (L1)* | 0 | 0 | 0 | -1 | 0 | 0 | 0 |
| *ND5* | -5 | -5 | -5 | 0 | 0 | -5 | -5 |
| *ND6* | 0 | 0 | -1 | 0 | 0 | 0 | 0 |
| *tRNA-Glu (E)* | 5 | 5 | 5 | 5 | 0 | 5 | 5 |
| *Cytb* | 0 | 0 | 0 | 0 | 0 | 0 | 0 |
| *tRNA-Thr (T)* | 3 | 2 | 3 | 3 | 3 | 3 | 3 |
| *tRNA-Pro (P)* | 0 | 0 | 0 | 0 | 0 | 0 | 0 |
| *Control region* | - | - | - | - | - | - | - |

**Table S3** The comprehensive comparison of the start and stop codons of the PCGs across seven *Chitala* mitogenomes.

| **Gene** | ***C. borneensis* (OR466573)** | | ***C. lopis* (OQ446559)** | | ***C. lopis* (AP008922)** | | ***C. chitala* (ON764424)** | | ***C. chitala* (KX894524)** | | ***C. ornata* (AP008923)** | | ***C. blanci* (AP008921)** | |
| --- | --- | --- | --- | --- | --- | --- | --- | --- | --- | --- | --- | --- | --- | --- |
|  | **Start** | **Stop** | **Start** | **Stop** | **Start** | **Stop** | **Start** | **Stop** | **Start** | **Stop** | **Start** | **Stop** | **Start** | **Stop** |
| *ND1* | ATG | TAA | ATG | TAA | ATG | TAA | ATG | TAA | ATG | TAA | ATG | TAA | ATG | TAA |
| *ND2* | ATG | TAG | ATG | T-- | ATG | T-- | ATG | TAG | ATG | T-- | ATG | T-- | ATG | T-- |
| *COI* | GTG | AGG | GTG | T-- | GTG | T-- | GTG | T-- | GTG | T-- | GTG | T-- | GTG | T-- |
| *COII* | ATG | AGA | ATG | T-- | ATG | T-- | ATG | T-- | ATG | T-- | ATG | T-- | ATG | T-- |
| *ATP8* | ATG | TAA | ATG | TAA | ATG | TAA | ATG | TAA | ATG | TAA | ATG | TAA | ATG | TAA |
| *ATP6* | ATG | TAA | ATG | TAA | ATG | TA- | ATG | TAA | ATG | TAA | ATG | TA- | ATG | TA- |
| *COIII* | ATG | TAA | ATG | TA- | ATG | TA- | ATG | TA- | ATG | A-- | ATG | TA- | ATG | TA- |
| *ND3* | ATG | TAG | ATG | T-- | ATG | T-- | ATG | T-- | ATG | T-- | ATG | T-- | ATG | T-- |
| *ND4L* | ATG | TAA | ATG | TAA | ATG | TAA | ATG | TAA | ATG | TAA | ATG | TAA | ATG | TAA |
| *ND4* | ATG | T-- | ATG | T-- | ATG | T-- | ATG | T-- | ATG | T-- | ATG | T-- | ATG | T-- |
| *ND5* | ATG | TAA | ATG | TAA | ATG | TAA | ATG | TAA | ATG | TAA | ATG | TAA | ATG | TAA |
| *ND6* | ATG | AGA | ATG | AGA | ATG | AGA | ATG | AGA | ATG | AGA | ATG | AGA | ATG | AGA |
| *Cytb* | ATG | T-- | ATG | T-- | ATG | T-- | ATG | T-- | ATG | - | ATG | T-- | ATG | T-- |

**Table S4** The Ka/Ks ratios of 13 PCGs in the mitogenomes of Asian notopterid species.

| **Gene** | ***ND1*** | ***ND2*** | ***COI*** | ***COII*** | ***ATP8*** | ***ATP6*** | ***COIII*** | ***ND3*** | ***ND4L*** | ***ND4*** | ***ND5*** | ***ND6*** | ***Cytb*** |
| --- | --- | --- | --- | --- | --- | --- | --- | --- | --- | --- | --- | --- | --- |
| 1 | 0.099077417 | 0.120500189 | 0.009988475 | 0.049008499 | 0.256663801 | 0.062828846 | 0.663855422 | 0.091419816 | 0.017789553 | 1.035064935 | 0.051289566 | 0.078205128 | 0.096327834 |
| 2 | 0.101002313 | 0.115661737 | 0.013817608 | 0.042195256 | 0.256663801 | 0.057814485 | 0.686746988 | 0.091419816 | 0.017789553 | 0.919335706 | 0.058258083 | 0.078205128 | 0.096327834 |
| 3 | 0.049041714 | 0.161891892 | 0.011460380 | 0.023468379 | 0.215836526 | 0.041486958 | 0.572572573 | 0.098570724 | 0.032913165 | 0.938086304 | 0.069719043 | 0.101146497 | 0.042421354 |
| 4 | 0.052296624 | 0.174078091 | 0.011460380 | 0.058378378 | 0.215836526 | 0.041486958 | 0.600836820 | 0.098570724 | 0.032994033 | 1.035372849 | 0.069719043 | 0.104117493 | 0.040472942 |
| 5 | 0.040578358 | 0.084763124 | 0.009451796 | 0.009783728 | 0.210289710 | 0.054286482 | 0.602122016 | 0.059550562 | 0.050627526 | 0.873508353 | 0.066096423 | 0.073184358 | 0.022775526 |
| 6 | 0.054853455 | 0.111339148 | 0.006430868 | 0.029970924 | 0.198896632 | 0.058993997 | 0.661852167 | 0.050608584 | 0.041999408 | 0.952338923 | 0.057625738 | 0.044767090 | 0.035147152 |
| 7 | 0.022639897 | 0.063768116 | 0.006743295 | 0.009527391 | 0.186201958 | 0.017742293 | 0.601365546 | 0.045684667 | 0.014308585 | 0.956109134 | 0.041391799 | 0.056451613 | 0.044061303 |
| 8 | 0.023923445 | 0.074881517 | 0.010207497 | 0.008720930 | 0.182758621 | 0.035510799 | 0.570977918 | 0.050974513 | 0.027669129 | 1.069236260 | 0.045990394 | 0.068248773 | 0.037328628 |
| 9 | 0.096079994 | 0.178929950 | 0.018420211 | 0.039355213 | 0.177093059 | 0.090681676 | 0.704375246 | 0.097643287 | 0.064229612 | 1.142697049 | 0.100016586 | 0.235746486 | 0.051596624 |
| 10 | 0.096079994 | 0.174462011 | 0.017844579 | 0.040471924 | 0.177093059 | 0.090681676 | 0.706161137 | 0.097643287 | 0.064229612 | 1.149962603 | 0.100209497 | 0.218436679 | 0.050718440 |
| 11 | 0.101004665 | 0.144865459 | 0.017914587 | 0.041567830 | 0.394701543 | 0.120266748 | 0.702613176 | 0.102630556 | 0.087635495 | 1.314272850 | 0.084409449 | 0.244915528 | 0.063849250 |
| 12 | 0.076109937 | 0.150592217 | 0.021399829 | 0.033437234 | 0.262459991 | 0.053534704 | 0.943089431 | 0.086908766 | 0.045209054 | 1.195101351 | 0.118618189 | 0.130052214 | 0.073336311 |
| **Average** | 0.067723984 | 0.129644454 | 0.012928292 | 0.032157141 | 0.227874602 | 0.060442969 | 0.668047370 | 0.080968775 | 0.041449561 | 1.048423860 | 0.071945318 | 0.119456416 | 0.054530266 |
| **STDEV** | 0.03062555 | 0.040552581 | 0.004905132 | 0.016308498 | 0.061091129 | 0.028077154 | 0.100845843 | 0.022205253 | 0.022279554 | 0.131203296 | 0.024103791 | 0.072273491 | 0.023532412 |

**Table S5** The abundance of amino acids and RSCU value of PCGs in *Chitala* species.

| ***Chitala borneensis* (OR466573)** | | | | |  |  |  |  |  |  |  |
| --- | --- | --- | --- | --- | --- | --- | --- | --- | --- | --- | --- |
| Codon | Count | RSCU | Codon | Count | RSCU | Codon | Count | RSCU | Codon | Count | RSCU |
| UUU(F) | 82 | 1.03 | UCU(S) | 75 | 1.25 | UAU(Y) | 88 | 1.04 | UGU(C) | 17 | 0.69 |
| UUC(F) | 78 | 0.98 | UCC(S) | 64 | 1.07 | UAC(Y) | 82 | 0.96 | UGC(C) | 32 | 1.31 |
| UUA(L) | 82 | 1.08 | UCA(S) | 95 | 1.58 | UAA(*) | 71 | 1.12 | UGA(*) | 55 | 0.86 |
| UUG(L) | 45 | 0.59 | UCG(S) | 24 | 0.40 | UAG(*) | 65 | 1.02 | UGG(W) | 22 | 1.00 |
| CUU(L) | 87 | 1.15 | CCU(P) | 111 | 1.31 | CAU(H) | 81 | 0.93 | CGU(R) | 17 | 0.61 |
| CUC(L) | 69 | 0.91 | CCC(P) | 84 | 0.99 | CAC(H) | 93 | 1.07 | CGC(R) | 34 | 1.21 |
| CUA(L) | 116 | 1.53 | CCA(P) | 115 | 1.35 | CAA(Q) | 82 | 1.28 | CGA(R) | 27 | 0.96 |
| CUG(L) | 56 | 0.74 | CCG(P) | 30 | 0.35 | CAG(Q) | 46 | 0.72 | CGG(R) | 25 | 0.89 |
| AUU(I) | 117 | 1.26 | ACU(T) | 100 | 1.11 | AAU(N) | 82 | 0.87 | AGU(S) | 35 | 0.58 |
| AUC(I) | 83 | 0.89 | ACC(T) | 89 | 0.99 | AAC(N) | 106 | 1.13 | AGC(S) | 67 | 1.12 |
| AUA(I) | 79 | 0.85 | ACA(T) | 139 | 1.55 | AAA(K) | 86 | 1.54 | AGA(R) | 26 | 0.93 |
| AUG(M) | 57 | 1.00 | ACG(T) | 31 | 0.35 | AAG(K) | 26 | 0.46 | AGG(R) | 39 | 1.39 |
| GUU(V) | 26 | 0.91 | GCU(A) | 35 | 0.74 | GAU(D) | 35 | 0.90 | GGU(G) | 27 | 0.84 |
| GUC(V) | 14 | 0.49 | GCC(A) | 86 | 1.81 | GAC(D) | 43 | 1.10 | GGC(G) | 37 | 1.16 |
| GUA(V) | 53 | 1.86 | GCA(A) | 61 | 1.28 | GAA(E) | 61 | 1.37 | GGA(G) | 45 | 1.41 |
| GUG(V) | 21 | 0.74 | GCG(A) | 8 | 0.17 | GAG(E) | 28 | 0.63 | GGG(G) | 19 | 0.59 |
| ***Chitala lopis* (OQ446559)** | | | |  |  |  |  |  |  |  |  |
| UUU(F) | 83 | 1.08 | UCU(S) | 73 | 1.15 | UAU(Y) | 78 | 0.95 | UGU(C) | 25 | 0.88 |
| UUC(F) | 71 | 0.92 | UCC(S) | 70 | 1.10 | UAC(Y) | 86 | 1.05 | UGC(C) | 32 | 1.12 |
| UUA(L) | 76 | 1.02 | UCA(S) | 98 | 1.54 | UAA(*) | 66 | 1.05 | UGA(*) | 61 | 0.97 |
| UUG(L) | 32 | 0.43 | UCG(S) | 34 | 0.54 | UAG(*) | 62 | 0.98 | UGG(W) | 23 | 1.00 |
| CUU(L) | 79 | 1.06 | CCU(P) | 112 | 1.32 | CAU(H) | 86 | 0.95 | CGU(R) | 14 | 0.53 |
| CUC(L) | 81 | 1.08 | CCC(P) | 85 | 1.00 | CAC(H) | 96 | 1.05 | CGC(R) | 31 | 1.17 |
| CUA(L) | 125 | 1.67 | CCA(P) | 113 | 1.33 | CAA(Q) | 89 | 1.37 | CGA(R) | 28 | 1.06 |
| CUG(L) | 55 | 0.74 | CCG(P) | 29 | 0.34 | CAG(Q) | 41 | 0.63 | CGG(R) | 21 | 0.79 |
| AUU(I) | 113 | 1.21 | ACU(T) | 92 | 1.05 | AAU(N) | 90 | 0.96 | AGU(S) | 33 | 0.52 |
| AUC(I) | 84 | 0.90 | ACC(T) | 87 | 0.99 | AAC(N) | 97 | 1.04 | AGC(S) | 73 | 1.15 |
| AUA(I) | 84 | 0.90 | ACA(T) | 139 | 1.58 | AAA(K) | 89 | 1.53 | AGA(R) | 26 | 0.98 |
| AUG(M) | 47 | 1.00 | ACG(T) | 34 | 0.39 | AAG(K) | 27 | 0.47 | AGG(R) | 39 | 1.47 |
| GUU(V) | 25 | 0.84 | GCU(A) | 44 | 0.89 | GAU(D) | 35 | 0.92 | GGU(G) | 15 | 0.50 |
| GUC(V) | 21 | 0.71 | GCC(A) | 83 | 1.69 | GAC(D) | 41 | 1.08 | GGC(G) | 44 | 1.47 |
| GUA(V) | 54 | 1.82 | GCA(A) | 62 | 1.26 | GAA(E) | 64 | 1.52 | GGA(G) | 41 | 1.37 |
| GUG(V) | 19 | 0.64 | GCG(A) | 8 | 0.16 | GAG(E) | 20 | 0.48 | GGG(G) | 20 | 0.67 |
| ***Chitala lopis* (AP008922)** | | | |  |  |  |  |  |  |  |  |
| Codon | Count | RSCU | Codon | Count | RSCU | Codon | Count | RSCU | Codon | Count | RSCU |
| UUU(F) | 84 | 1.09 | UCU(S) | 75 | 1.19 | UAU(Y) | 78 | 0.95 | UGU(C) | 25 | 0.88 |
| UUC(F) | 70 | 0.91 | UCC(S) | 68 | 1.08 | UAC(Y) | 86 | 1.05 | UGC(C) | 32 | 1.12 |
| UUA(L) | 75 | 1.00 | UCA(S) | 98 | 1.56 | UAA(*) | 67 | 1.05 | UGA(*) | 61 | 0.96 |
| UUG(L) | 32 | 0.43 | UCG(S) | 33 | 0.52 | UAG(*) | 63 | 0.99 | UGG(W) | 23 | 1.00 |
| CUU(L) | 78 | 1.04 | CCU(P) | 110 | 1.30 | CAU(H) | 87 | 0.95 | CGU(R) | 14 | 0.53 |
| CUC(L) | 80 | 1.07 | CCC(P) | 86 | 1.01 | CAC(H) | 96 | 1.05 | CGC(R) | 31 | 1.17 |
| CUA(L) | 128 | 1.71 | CCA(P) | 112 | 1.32 | CAA(Q) | 88 | 1.38 | CGA(R) | 28 | 1.06 |
| CUG(L) | 56 | 0.75 | CCG(P) | 31 | 0.37 | CAG(Q) | 40 | 0.63 | CGG(R) | 21 | 0.79 |
| AUU(I) | 113 | 1.20 | ACU(T) | 92 | 1.05 | AAU(N) | 89 | 0.95 | AGU(S) | 33 | 0.52 |
| AUC(I) | 84 | 0.89 | ACC(T) | 87 | 0.99 | AAC(N) | 99 | 1.05 | AGC(S) | 71 | 1.13 |
| AUA(I) | 86 | 0.91 | ACA(T) | 138 | 1.57 | AAA(K) | 90 | 1.55 | AGA(R) | 26 | 0.98 |
| AUG(M) | 47 | 1.00 | ACG(T) | 34 | 0.39 | AAG(K) | 26 | 0.45 | AGG(R) | 39 | 1.47 |
| GUU(V) | 25 | 0.85 | GCU(A) | 43 | 0.87 | GAU(D) | 35 | 0.92 | GGU(G) | 15 | 0.50 |
| GUC(V) | 21 | 0.72 | GCC(A) | 84 | 1.71 | GAC(D) | 41 | 1.08 | GGC(G) | 44 | 1.47 |
| GUA(V) | 52 | 1.78 | GCA(A) | 62 | 1.26 | GAA(E) | 65 | 1.55 | GGA(G) | 40 | 1.33 |
| GUG(V) | 19 | 0.65 | GCG(A) | 8 | 0.16 | GAG(E) | 19 | 0.45 | GGG(G) | 21 | 0.70 |
| ***Chitala chitala* (ON764424)** | | | |  |  |  |  |  |  |  |  |
| Codon | Count | RSCU | Codon | Count | RSCU | Codon | Count | RSCU | Codon | Count | RSCU |
| UUU(F) | 84 | 1.08 | UCU(S) | 80 | 1.28 | UAU(Y) | 78 | 0.95 | UGU(C) | 20 | 0.69 |
| UUC(F) | 72 | 0.92 | UCC(S) | 72 | 1.15 | UAC(Y) | 86 | 1.05 | UGC(C) | 38 | 1.31 |
| UUA(L) | 75 | 0.99 | UCA(S) | 90 | 1.44 | UAA(*) | 68 | 1.09 | UGA(*) | 59 | 0.94 |
| UUG(L) | 36 | 0.47 | UCG(S) | 34 | 0.54 | UAG(*) | 61 | 0.97 | UGG(W) | 29 | 1.00 |
| CUU(L) | 92 | 1.21 | CCU(P) | 103 | 1.26 | CAU(H) | 82 | 0.95 | CGU(R) | 21 | 0.76 |
| CUC(L) | 74 | 0.98 | CCC(P) | 78 | 0.95 | CAC(H) | 90 | 1.05 | CGC(R) | 31 | 1.13 |
| CUA(L) | 123 | 1.62 | CCA(P) | 116 | 1.41 | CAA(Q) | 92 | 1.38 | CGA(R) | 24 | 0.87 |
| CUG(L) | 55 | 0.73 | CCG(P) | 31 | 0.38 | CAG(Q) | 41 | 0.62 | CGG(R) | 21 | 0.76 |
| AUU(I) | 116 | 1.20 | ACU(T) | 95 | 1.10 | AAU(N) | 86 | 0.90 | AGU(S) | 27 | 0.43 |
| AUC(I) | 84 | 0.87 | ACC(T) | 88 | 1.01 | AAC(N) | 105 | 1.10 | AGC(S) | 72 | 1.15 |
| AUA(I) | 89 | 0.92 | ACA(T) | 134 | 1.54 | AAA(K) | 87 | 1.60 | AGA(R) | 24 | 0.87 |
| AUG(M) | 55 | 1.00 | ACG(T) | 30 | 0.35 | AAG(K) | 22 | 0.40 | AGG(R) | 44 | 1.60 |
| GUU(V) | 29 | 1.02 | GCU(A) | 37 | 0.79 | GAU(D) | 30 | 0.76 | GGU(G) | 26 | 0.83 |
| GUC(V) | 16 | 0.56 | GCC(A) | 79 | 1.68 | GAC(D) | 49 | 1.24 | GGC(G) | 39 | 1.24 |
| GUA(V) | 55 | 1.93 | GCA(A) | 61 | 1.30 | GAA(E) | 59 | 1.39 | GGA(G) | 45 | 1.43 |
| GUG(V) | 14 | 0.49 | GCG(A) | 11 | 0.23 | GAG(E) | 26 | 0.61 | GGG(G) | 16 | 0.51 |
| ***Chitala chitala* (KX894524)** | | | |  |  |  |  |  |  |  |  |
| Codon | Count | RSCU | Codon | Count | RSCU | Codon | Count | RSCU | Codon | Count | RSCU |
| UUU(F) | 96 | 1.07 | UCU(S) | 66 | 1.22 | UAU(Y) | 80 | 0.98 | UGU(C) | 11 | 0.63 |
| UUC(F) | 83 | 0.93 | UCC(S) | 79 | 1.46 | UAC(Y) | 84 | 1.02 | UGC(C) | 24 | 1.37 |
| UUA(L) | 107 | 1.33 | UCA(S) | 79 | 1.46 | UAA(*) | 66 | 1.00 | UGA(*) | 73 | 1.11 |
| UUG(L) | 44 | 0.55 | UCG(S) | 25 | 0.46 | UAG(*) | 59 | 0.89 | UGG(W) | 16 | 1.00 |
| CUU(L) | 79 | 0.98 | CCU(P) | 74 | 1.03 | CAU(H) | 59 | 0.70 | CGU(R) | 25 | 1.01 |
| CUC(L) | 72 | 0.89 | CCC(P) | 77 | 1.07 | CAC(H) | 110 | 1.30 | CGC(R) | 33 | 1.33 |
| CUA(L) | 143 | 1.77 | CCA(P) | 110 | 1.53 | CAA(Q) | 126 | 1.47 | CGA(R) | 27 | 1.09 |
| CUG(L) | 39 | 0.48 | CCG(P) | 27 | 0.38 | CAG(Q) | 45 | 0.53 | CGG(R) | 21 | 0.85 |
| AUU(I) | 118 | 1.13 | ACU(T) | 62 | 0.78 | AAU(N) | 63 | 0.88 | AGU(S) | 27 | 0.50 |
| AUC(I) | 97 | 0.93 | ACC(T) | 101 | 1.28 | AAC(N) | 80 | 1.12 | AGC(S) | 48 | 0.89 |
| AUA(I) | 97 | 0.93 | ACA(T) | 134 | 1.70 | AAA(K) | 87 | 1.61 | AGA(R) | 21 | 0.85 |
| AUG(M) | 39 | 1.00 | ACG(T) | 19 | 0.24 | AAG(K) | 21 | 0.39 | AGG(R) | 22 | 0.89 |
| GUU(V) | 38 | 1.16 | GCU(A) | 50 | 0.82 | GAU(D) | 35 | 0.79 | GGU(G) | 20 | 0.55 |
| GUC(V) | 26 | 0.79 | GCC(A) | 98 | 1.61 | GAC(D) | 54 | 1.21 | GGC(G) | 49 | 1.35 |
| GUA(V) | 56 | 1.71 | GCA(A) | 85 | 1.40 | GAA(E) | 61 | 1.34 | GGA(G) | 51 | 1.41 |
| GUG(V) | 11 | 0.34 | GCG(A) | 10 | 0.16 | GAG(E) | 30 | 0.66 | GGG(G) | 25 | 0.69 |
| ***Chitala ornata* (AP008923)** | | | |  |  |  |  |  |  |  |  |
| Codon | Count | RSCU | Codon | Count | RSCU | Codon | Count | RSCU | Codon | Count | RSCU |
| UUU(F) | 84 | 1.08 | UCU(S) | 80 | 1.28 | UAU(Y) | 78 | 0.95 | UGU(C) | 20 | 0.69 |
| UUC(F) | 72 | 0.92 | UCC(S) | 72 | 1.15 | UAC(Y) | 86 | 1.05 | UGC(C) | 38 | 1.31 |
| UUA(L) | 75 | 0.99 | UCA(S) | 90 | 1.44 | UAA(*) | 68 | 1.09 | UGA(*) | 59 | 0.94 |
| UUG(L) | 36 | 0.47 | UCG(S) | 34 | 0.54 | UAG(*) | 61 | 0.97 | UGG(W) | 29 | 1.00 |
| CUU(L) | 92 | 1.21 | CCU(P) | 103 | 1.26 | CAU(H) | 82 | 0.95 | CGU(R) | 21 | 0.76 |
| CUC(L) | 74 | 0.98 | CCC(P) | 78 | 0.95 | CAC(H) | 90 | 1.05 | CGC(R) | 31 | 1.13 |
| CUA(L) | 123 | 1.62 | CCA(P) | 116 | 1.41 | CAA(Q) | 92 | 1.38 | CGA(R) | 24 | 0.87 |
| CUG(L) | 55 | 0.73 | CCG(P) | 31 | 0.38 | CAG(Q) | 41 | 0.62 | CGG(R) | 21 | 0.76 |
| AUU(I) | 116 | 1.20 | ACU(T) | 95 | 1.10 | AAU(N) | 86 | 0.90 | AGU(S) | 27 | 0.43 |
| AUC(I) | 84 | 0.87 | ACC(T) | 88 | 1.01 | AAC(N) | 105 | 1.10 | AGC(S) | 72 | 1.15 |
| AUA(I) | 89 | 0.92 | ACA(T) | 134 | 1.54 | AAA(K) | 87 | 1.60 | AGA(R) | 24 | 0.87 |
| AUG(M) | 55 | 1.00 | ACG(T) | 30 | 0.35 | AAG(K) | 22 | 0.40 | AGG(R) | 44 | 1.60 |
| GUU(V) | 29 | 1.02 | GCU(A) | 37 | 0.79 | GAU(D) | 30 | 0.76 | GGU(G) | 26 | 0.83 |
| GUC(V) | 16 | 0.56 | GCC(A) | 79 | 1.68 | GAC(D) | 49 | 1.24 | GGC(G) | 39 | 1.24 |
| GUA(V) | 55 | 1.93 | GCA(A) | 61 | 1.30 | GAA(E) | 59 | 1.39 | GGA(G) | 45 | 1.43 |
| GUG(V) | 14 | 0.49 | GCG(A) | 11 | 0.23 | GAG(E) | 26 | 0.61 | GGG(G) | 16 | 0.51 |
| ***Chitala blanci* (AP008921)** | | | |  |  |  |  |  |  |  |  |
| Codon | Count | RSCU | Codon | Count | RSCU | Codon | Count | RSCU | Codon | Count | RSCU |
| UUU(F) | 88 | 1.14 | UCU(S) | 65 | 1.07 | UAU(Y) | 92 | 0.99 | UGU(C) | 20 | 0.68 |
| UUC(F) | 67 | 0.86 | UCC(S) | 70 | 1.15 | UAC(Y) | 93 | 1.01 | UGC(C) | 39 | 1.32 |
| UUA(L) | 76 | 1.02 | UCA(S) | 96 | 1.58 | UAA(*) | 62 | 1.01 | UGA(*) | 62 | 1.01 |
| UUG(L) | 38 | 0.51 | UCG(S) | 30 | 0.49 | UAG(*) | 60 | 0.98 | UGG(W) | 26 | 1.00 |
| CUU(L) | 82 | 1.11 | CCU(P) | 109 | 1.31 | CAU(H) | 76 | 0.94 | CGU(R) | 24 | 0.87 |
| CUC(L) | 66 | 0.89 | CCC(P) | 82 | 0.98 | CAC(H) | 86 | 1.06 | CGC(R) | 29 | 1.05 |
| CUA(L) | 126 | 1.70 | CCA(P) | 112 | 1.35 | CAA(Q) | 85 | 1.27 | CGA(R) | 27 | 0.98 |
| CUG(L) | 57 | 0.77 | CCG(P) | 30 | 0.36 | CAG(Q) | 49 | 0.73 | CGG(R) | 18 | 0.65 |
| AUU(I) | 119 | 1.21 | ACU(T) | 94 | 1.10 | AAU(N) | 88 | 0.96 | AGU(S) | 29 | 0.48 |
| AUC(I) | 79 | 0.81 | ACC(T) | 86 | 1.01 | AAC(N) | 95 | 1.04 | AGC(S) | 75 | 1.23 |
| AUA(I) | 96 | 0.98 | ACA(T) | 135 | 1.58 | AAA(K) | 88 | 1.54 | AGA(R) | 28 | 1.02 |
| AUG(M) | 54 | 1.00 | ACG(T) | 27 | 0.32 | AAG(K) | 26 | 0.46 | AGG(R) | 39 | 1.42 |
| GUU(V) | 32 | 1.06 | GCU(A) | 47 | 0.96 | GAU(D) | 33 | 0.89 | GGU(G) | 26 | 0.79 |
| GUC(V) | 15 | 0.50 | GCC(A) | 80 | 1.63 | GAC(D) | 41 | 1.11 | GGC(G) | 42 | 1.27 |
| GUA(V) | 55 | 1.82 | GCA(A) | 61 | 1.24 | GAA(E) | 57 | 1.41 | GGA(G) | 37 | 1.12 |
| GUG(V) | 19 | 0.63 | GCG(A) | 8 | 0.16 | GAG(E) | 24 | 0.59 | GGG(G) | 27 | 0.82 |
